# Cation-Induced Structural Diversity in the Cobalt(II)/Nitranilato System: A Monomer, a Dimer, a Trimer and a Chain

**DOI:** 10.1021/acs.cgd.5c00210

**Published:** 2025-03-24

**Authors:** Cristina Pintado-Zaldo, Louise Bureller, Gonzalo de Joz-Latorre, Carlos J. Gómez-García, Samia Benmansour

**Affiliations:** Departamento de Química Inorgánica, Universidad de Valencia, Dr. Moliner 50, 46100 Burjasot, Spain

## Abstract

Herein, we show how it is possible to prepare a monomer, a dimer, a trimer and a chain with cobalt­(II) and the ligand nitranilato (3,6-dinitro-2,5-dihydroxy-1,4-benzoquinone dianion = C_6_O_4_(NO_2_)_2_^2–^ = NA^2–^) by simply changing the counter-cations. Thus, we show that when we combine the ligand NA^2–^ with cobalt­(II) in the presence of a bulky cation as PPh_4_^+^, we obtain compound (PPh_4_)_4_[Co­(NA)_3_] (**1**), which contains the first monomeric anion of the type [Co­(L)_3_]^4–^ (L = any anilato ligand). With a smaller cation as NMe_4_^+^, we obtain (NMe_4_)_2_[Co_2_(NA)_3_(H_2_O)_4_] (**2**), a rare example of anilato-containing dimer based on a transition metal (TM). With a larger cation as NPr_4_^+^, we obtain a trinuclear cobalt–nitranilato complex formulated as (NPr_4_)_2_[Co_3_(NA)_4_(H_2_O)_6_] (**3**) and finally, when using the small NH_4_^+^ cation, we obtain the neutral chain: [Co­(NA)­(H_2_O)_2_] (**4**). This work presents the synthesis and structure of these compounds and the magnetic characterization of compounds **2**–**4** that show the presence of very weak antiferromagnetic interactions between the cobalt centers through the anilato bridges.

## Graphical abstract

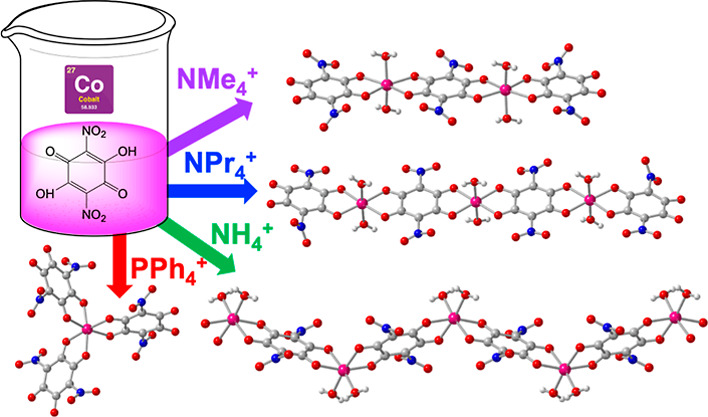

## Introduction

The synthesis and study of coordination compounds and polymers with metal ions and anilato-type ligands (derivatives of the 2,5-dihydroxy-1,4-benzoquinone) constitute an area of growing interest in materials science and coordination chemistry. Thus, the number of reported and studied metal complexes with anilato ligands has experienced a huge increase in the past decade.^1–4^

Although initially anilato ligands were mainly combined with transition metal ions and s-block metals, many anilato-based lattices with lanthanoid metal ions have also been reported in the last two decades.^1,5–12^

The most common anilato ligands used to date are the 3,6-derivatives of the 2,5-dihydroxy-1,4-benzoquinone, (H_2_C_6_O_4_X_2_, Scheme 1) with X = Cl and Br (chloranilic and bromanilic acids, respectively).^1–3^ Nevertheless, in the last years, other derivatives with X = H, F, I, NO_2_, CH_3_ and even asymmetric ones, with X = Cl/CN or Cl/NO_2_, have been used to prepare discrete complexes and extended lattices.^1–3,12,13^

**Scheme 1:**
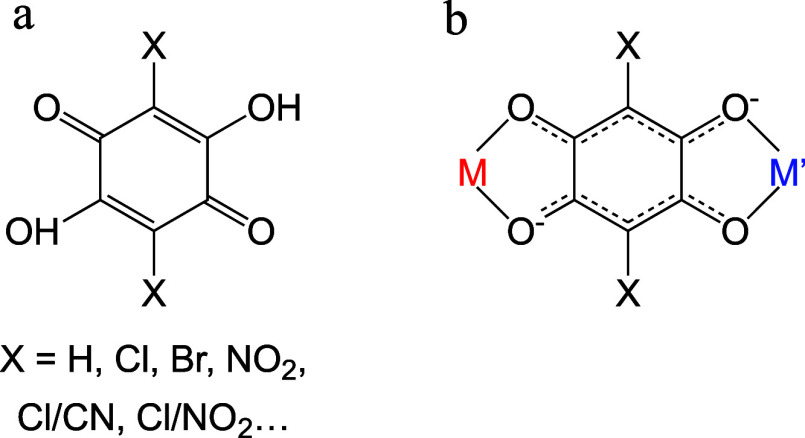
Anilato Type Ligands and Their Bis-bidentate Coordination Mode

If we focus on the ligand nitranilato (X = NO_2_), only 13 compounds, where the nitranilato ligand is coordinated to any metal ion (Table 1), can be found in the CCDC in February 2025. These 13 compounds include: two monomers, three dimers, one tetramer, five chains, one 2D and one 3D extended lattices. If we look at the metal ions, we can see that there are five compounds with Fe, two with Ag and one with Zn, Mo, Mn, Cr, Cu and Co. Except in the two monomers and one dimer (where the nitranilato ligand acts as bidentate terminal) and in the tetramer (where it acts as tetrakis-monodentate), the nitranilato ligand acts as a bis-bidentate bridge (Scheme 1) in the nine remaining reported compounds (Table 1).

**Table 1:** All the Reported Nitranilato-Based Compounds with Any Transition Metal

CCDC	formula	structure	NA	ref
BEPHUC	[Fe_2_(μ-O)(NA)_2_(C_18_H_18_N_4_)_2_]	dimer	terminal	^14^
FATTEZ	[Zn(NA)(H_2_O)_2_]	1D-zz	bridge	^15^
LEJBEI	[Mo_4_(NA)(C_15_H_15_N_2_O_2_)_6_]·5H_2_O	tetramer	bridge	^16^
LUNFEI	[Fe_2_(NA)_2_(C_21_H_24_N_4_)](BF_4_)_2_·4CH_3_CN	dimer	bridge	^17^
LUNFIM	[Fe_2_(NA)_2_(C_21_H_24_N_4_)](BF_4_)	dimer	bridge	^17^
LUYHIW	[Mn(NA)(C_15_H_11_N_3_)]	1D-zz	bridge	^18^
RADBIK	(PBu_4_)_3_[Cr(NA)_3_]	monomer	terminal	^19^
ROJKEJ	Ag_2_(NA)	3D	bridge	^13^
ROJKIN	(NMe_2_H_2_)[Ag(NA)]	1D-lad	bridge	^13^
ROJKOT	[Cu(NA)(H_2_O)_2_]	1D-zz	bridge	^13^
ROJLAG	[Co(NA)(H_2_O)_2_]	1D-zz	bridge	^13^
RUYXIU	(PBu_4_)_3_[Fe(NA)_3_]	Monomer	terminal	^19^
YEGWUF	(C_16_H_17_N_2_)_2_[FeNa(NA)_3_]·CH_3_CN	2D	bridge	^20^

The two reported monomers are isostructural and contain isolated and chiral tris­(nitranilato)­metalate [M­(NA)_3_]^3–^ anions with M = Cr^III^ (RADBIK) and Fe^III^ (RUYXIU). Interestingly, in both complexes, the charge-compensating cations are bulky PBu_4_^+^ cations.^19^

One of the three dimers is an oxido-bridged Fe^III^ dimer: [Fe_2_(μ-O)­(NA)_2_(L)_2_] (BEPHUC) with L = C_18_H_18_N_4_ = tris­(2-pyridylmethyl)­amine), where both Fe^III^ centers are coordinated to a bidentate nitranilato terminal ligand, a bridging O atom and a terminal tris­(2-pyridylmethyl)­amine to complete their octahedral coordination environment.^14^ There are also two closely related dimers, formulated as [Fe_2_(NA)_2_(L)]­(BF_4_)_2_·4CH_3_CN (LUNFEI) and [Fe_2_(NA)_2_(L)]­(BF_4_) (LUNFIM) with L = C_21_H_24_N_4_ = *N*,*N*,*N*-tris­[(6-methylpyridin-2-yl)­methyl]­amine, where the two Fe centers are connected through a bis-bidentate nitranilato ligand and complete their octahedral coordination with a capping tetradentate *N*,*N*,*N*-tris­[(6-methylpyridin-2-yl)­methyl]­amine ligand. Both dimers show the same structure. The only difference between them is that the second one has been reduced by one electron and it has a +1 charge whereas the first one has a +2 charge.^17^

The only reported tetramer, formulated as [Mo_4_(NA)­(L)_6_]·5H_2_O (LEJBEI) (with L = C_15_H_15_N_2_O_2_ = *N*,*N*′-bis­(4-methoxyphenyl)­formamidinato-*N*,*N*′) contains two Mo-dimers with a Mo–Mo bond connected through an original tetrakis monodentate bridging nitranilato ligand (each O atom on the nitranilato ring is coordinated to a different Mo atom). Each Mo_2_ dimer is coordinated to three *N*,*N*′-bis­(4-methoxyphenyl)­formamidinato-*N*,*N*′ ligands and the Mo atoms present a square planar coordination geometry (i.e., a square pyramid when considering the other Mo atom).^16^

Three of the five chain compounds are closely related. They are formulated as [M­(NA)­(H_2_O)_2_] with M = Zn^II^ (FATTEZ), Cu^II^ (ROJKOT) and Co^II^ (ROJLAG) and contain M^II^ centers coordinated to two bis-bidentate bridging nitranilato ligands and to two cis water molecules, forming zigzag chains.^13,15^ There is a Mn^II^ chain formulated as [Mn­(NA)­(L)] (LUYHIW) with L = C_15_H_11_N_3_ = terpy = 2,2′:6′,2″-terpyridine, that contains Mn^II^ ions bridged by bis-bidentate nitranilato ligands forming zigzag chains. The Mn^II^ ions complete their heptacoordination environment with a tridentate terpy ligand.^18^ The last chain compound is (NMe_2_H_2_)­[Ag­(NA)] (ROJKIN), that contains double anionic ladder-type [Ag­(NA)]^−^ chains with NMe_2_H_2_^+^ cations located between these chains.^13^

There is also a 2D structure formulated as (DAMS)_2_[FeNa­(NA)_3_]·CH_3_CN (YEGWUF) where DAMS^+^ = C_16_H_17_N_2_^+^ = 4-[4-(dimethylamino)-α-styryl]-1-methylpyridinium. This original compound contains anionic layers formulated as [NaFe­(NA)_3_]^2–^ where the nitranilato ligands form honeycomb hexagonal layers with a Fe^III^ center and two Na^+^ ions alternating in the vertices of the hexagons. Additionally, a [Fe­(NA)_3_]^3–^ anion is occupying the center of the hexagons connecting the Na-containing vertices.^20^

Finally, there is a 3D lattice formulated as Ag_2_(NA) (ROJKEJ) where the nitranilato ligand is coordinated to a total of ten silver ions through its eight O atoms, resulting in a 3D lattice with a 32c topology.^13^

Besides the 2D lattice (DAMS)_2_[FeNa­(NA)_3_]·CH_3_CN (YEGWUF), where the nitranilato ligand is connected to Na^+^ and Fe^3+^ cations, there are also six compounds where the nitranilato ligand is coordinated to s-block metals (Table 2). These six nitranilato compounds with s-block metals are all polymers, due to the ability of s-block metals to semicoordinate to many donor atoms, resulting in high coordination numbers and high connectivity. Among these six compounds, two are chains, formulated as [M­(NA)­(H_2_O)_4_] with M = Ca (FIBVAN) and Sr (FIBVER).^21^ These compounds contain zigzag chains formed by bis-bidentate nitranilato ligands connecting Ca^2+^ or Sr^2+^ cations, as observed in the chain compounds [M­(NA)­(H_2_O)_2_] with M = Zn^II^, Cu^II^ and Co^II^ described above. The only difference is that now the Ca and Sr ions have coordination numbers of eight and complete their coordination environment with four additional coordinated water molecules instead of two, as observed for Zn, Cu and Co derivatives.^13,15^

**Table 2:** All the Reported Nitranilato-Based Compounds with s-Block Metals

CCDC	formula	structure	NA	ref
FIBVAN	Ca(NA)(H_2_O)_4_	1D	bridge	^21^
FIBVER	Sr(NA)(H_2_O)_4_	1D	bridge	^21^
ROJKUZ	[Fe(bpy)_3_][Na_2_(NA)_3_]·C_10_H_8_N_2_·5H_2_O	2D	bridge	^13^
ROJLEK	Na[Fe(bpy)_3_][Na(NA)_3_]·6H_2_O	2D	bridge	^13^
WIMPAJ	K_2_(NA)	3D	bridge	^22^
WIMPEN	Na_2_(NA)	3D	bridge	^22^
YEGWUF	(C_16_H_17_N_2_)_2_[FeNa(NA)_3_]·CH_3_CN	2D	bridge	^20^

There are also two closely related 2D lattices with nitranilato and s-block metals. The first, formulated as [Fe­(L)_3_]_2_[Na_2_(NA)_3_]·L·5H_2_O (ROJKUZ) (with L = C_10_H_8_N_2_ = bpy = bipyridine) contains anionic hexagonal honeycomb layers of the type [Na_2_(NA)_3_]^4–^ where the Na^+^ occupy the vertices and the nitranilato ligands form the sides of the hexagons. The [Fe­(bpy)_3_]^2+^ cations occupy the interlayer space.^13^ The other related 2D lattice with nitranilato and s-block metals is formulated as Na­[Fe­(L)_3_]­[Na­(NA)_3_]·6H_2_O (ROJLEK) (with L = C_10_H_8_N_2_ = bpy = bipyridine). In this compound, the Na^+^ cations are connected through nitranilato ligands to form rhombic layers parallel to the *ab* plane.^13^

Finally, there are two 3D lattices with nitranilato and s-block metals, formulated as M_2_(NA) with M = Na (WIMPEN) and K (WIMPAJ).^22^ These closely related lattices contain nitranilato ligands coordinated to up to six Na^+^ and ten K^+^ ions, respectively, using their eight O atoms, giving rise to two 3D lattices.

Although less common, nitranilato has also been used with lanthanoid ions in the series of five isostructural dimers formulated as [Ln_2_(NA)_3_(H_2_O)_10_]·6H_2_O with Ln = Gd^III^, Tb^III^, Dy^III^, Ho^III^ and Sm^III^ (Table 3).^23^ These dimers contain a bis-bidentate bridging nitranilato ligand connecting the two Ln^III^ ions that also present a terminal bidentate nitranilato ligand each and complete their nona-coordination with five water molecules.

**Table 3:** All the Reported Nitranilato-Based Compounds with Lanthanoids

CCDC	formula	structure	NA	ref
EDEZAR	[Gd_2_(NA)_3_(H_2_O)_10_]·6H_2_O	dimer	Bridge + terminal	^23^
EDEZEV	[Tb_2_(NA)_3_(H_2_O)_10_]·6H_2_O	dimer	Bridge + terminal	^23^
EDEZIZ	[Dy_2_(NA)_3_(H_2_O)_10_]·6H_2_O	dimer	Bridge + terminal	^23^
EDEZOV	[Ho_2_(NA)_3_(H_2_O)_10_]·6H_2_O	dimer	Bridge + terminal	^23^
EDEZUL	[Sm_2_(NA)_3_(H_2_O)_10_]·6H_2_O	dimer	Bridge + terminal	^23^

Interestingly, among the 24 reported nitranilato compounds with any metal (Tables 1–3) there is only one compound with cobalt: [Co­(NA)­(H_2_O)_2_] (ROJLAG, described above), that contains Co^II^ centers coordinated to two bis-bidentate bridging nitranilato ligands and to two cis water molecules, forming zigzag chains.^13^ The lack of more reported examples with nitranilato and cobalt is quite surprising because there are more than 70 reported compounds in the CCDC database containing cobalt with other anilato ligands (mainly C_6_O_4_H_2_^2–^ and C_6_O_4_Cl_2_^2–^).

Among these more than 70 cobalt-anilato compounds, there is only one monomer and four chain compounds containing only anilato ligands and water molecules. This monomer, formulated as (A)_2_[Co­(C_6_O_4_Cl_2_)_2_(H_2_O)_2_] (DOVHIK) with A = 3-Hydroxypyridinium, contains a Co^II^ center coordinated to two bidentate terminal chloranilate ligands (X = Cl) and two trans H_2_O molecules. The −2 charge of this anion is balanced by two 3-hydroxypyridinium cations.^24^ Two of the four chains are linear and are formulated as [Co­(C_6_O_4_H_2_)­(H_2_O)_2_]·0.5H_2_O^25^ (FULLEE) and [Co­(C_6_O_4_Cl_2_)­(H_2_O)_2_]·phz (KIQKEA) (phz = phenazine = C_12_H_8_N_2_).^26^ These chains are formed by bis-bidentate bridging anilato ligands connecting the Co^II^ ions that complete their octahedral coordination environment with two trans H_2_O molecules in the axial positions. The other two cobalt/anilato chains are zigzag chains since now the two water molecules occupy cis positions, as observed in [Co­(C_6_O_4_Cl_2_)­(H_2_O)_2_]·H_2_O (KIQJOJ)^26^ and in the previously described chain with nitranilato: [Co­(NA)­(H_2_O)_2_] (ROJLAG).^13^ Surprisingly, there are no reported cobalt/anilato dimers or trimers to date.

These results prompted us to investigate the synthesis and study of the crystallization conditions of novel complexes and lattices with cobalt and the nitranilato ligand. Furthermore, since we (and others) have observed the key role that may play in the final structure the counter-cations when the formed complexes are anions,^19,27^ we have decided to investigate the synthesis of novel complexes in the cobalt/nitranilato system using different counter-cations with different sizes as NH_4_^+^, NMe_4_^+^, NEt_4_^+^, NPr_4_^+^, NBu_4_^+^ and PPh_4_^+^. With this strategy, we have prepared four different compounds with nitranilato and cobalt­(II) using different cations. Thus, with the bulky cation PPh_4_^+^, we obtain compound (PPh_4_)_4_[Co­(NA)_3_] (**1**), which contains the first monomeric tetra-anion of the type [M^II^(L)_3_]^4–^ (L = any anilato ligand). With a smaller cation as NMe_4_^+^, we obtain (NMe_4_)_2_[Co_2_(NA)_3_(H_2_O)_4_] (**2**), a rare example of anilato-containing dimer. In contrast, with a larger cation as NPr_4_^+^, we obtain a trinuclear cobalt–nitranilato complex formulated as (NPr_4_)_2_[Co_3_(NA)_4_(H_2_O)_6_] (**3**) and finally, when using the small NH_4_^+^ cation, we obtain the neutral chain: [Co­(NA)­(H_2_O)_2_] (**4**), which is similar to the previously described [Co­(NA)­(H_2_O)_2_] (ROJLAG) chain.^13^ Here we present the synthesis and structure of these compounds and the magnetic characterization of compounds **2**–**4** that show the presence of very weak antiferromagnetic interactions between the cobalt centers through the anilato bridges.

## Experimental Section

### Starting Materials

All the chemicals and solvents were of reagent grade and used as received from commercial sources without further purification.

Given the limited number of crystals obtained in all cases, we checked the unit cell parameters of at least ten single crystals for each compound. We verified that all the crystals used for the magnetic characterization of each compound had the same color and shape. On the other hand, the phase purity was verified with powder X-ray diffraction for compounds **2–4** (Figures S1–S3, respectively).

### Synthesis of Na_2_(NA)·2H_2_O

The sodium nitranilato salt is obtained according to the method described in the literature^28^ with slight modifications as follows: a solution of sodium nitrite (NaNO_2_) (1.24 g; 18 mmol) in 10 mL of water is added, dropwise, to a solution of tetrachloro-1,4-benzoquinone (C_6_O_2_Cl_4_) (0.98 g; 4.0 mmol) in 100 mL of acetone, in a 250 mL round-bottom flask. The resulting mixture is refluxed with stirring for 1 h. A yellow-orange crystalline precipitate is obtained, filtered and air-dried (yield = 0.835 g, 76 %).

### Synthesis of (PPh_4_)_4_[Co­(NA)_3_] (**1**)

A clear yellow solution containing the salt Na_2_(NA)·2H_2_O (12.5 mg; 0.04 mmol), LiCl (8.5 mg; 0.2 mmol) and (PPh_4_)Br (83.8 mg; 0.2 mmol) in 7.5 mL of a H_2_O/MeOH (2:1) mixture, is added, dropwise, to a pink solution of CoCl_2_·6H_2_O (47.4 mg; 0.2 mmol) in 5 mL of MeOH under stirring. The solution, initially yellow, turns quickly to orange. The final solution is stirred for 20 min and left to evaporate at room temperature covered by parafilm with a few small pinholes. After 1 week, a few orange prism-shaped crystals, suitable for single crystal X-ray diffraction, were obtained, filtered and air-dried. Given the limited number of crystals obtained of compound **1**, we checked the unit cell parameters of at least ten single crystals of this compound but could not perform powder X-ray diffraction.

### Synthesis of (NMe_4_)_2_[Co_2_(NA)_3_(H_2_O)_4_] (**2**)

A clear yellow solution containing the salt Na_2_(NA)·2H_2_O (93.0 mg; 0.3 mmol) in 15 mL of H_2_O is added, dropwise, over a pink solution containing Co­(CH_3_COO)_2_·4H_2_O (39.8 mg; 0.16 mmol), LiCl (6.7 mg; 0.16 mmol) and (NMe_4_)Br (24.6 mg; 0.16 mmol) in 10 mL of H_2_O under stirring. The final solution is further stirred for 20 min and left to evaporate at room temperature covered by parafilm with a few small pinholes. After 9 days, shiny orange hexagonal crystals, suitable for single crystal X-ray diffraction, were obtained, filtered and air-dried (yield = 5.5 mg, 6.6 %). The phase purity of this compound is confirmed by powder X-ray diffraction analysis (Figure S1).

### Synthesis of (NPr_4_)_2_[Co_3_(NA)_4_(H_2_O)_6_] (**3**)

This compound is prepared by carefully layering in a thin tube two solutions with a buffer layer of a H_2_O/acetone (1:1) mixture separating both solutions. The bottom light yellow solution contains Na_2_(NA)·2H_2_O (37.2 mg; 0.12 mmol) dissolved in 5 mL of H_2_O. The blue top solution contains CoCl_2_·6H_2_O (19.0 mg, 0.08 mmol), LiCl (3.4 mg; 0.08 mmol) and (NPr_4_)Br (85.2 mg; 0.32 mmol) in 4 mL of acetone. The tube is sealed with parafilm and left undisturbed at room temperature. After one month, orange plate-like crystals, suitable for single crystal X-ray diffraction were obtained, filtered and air-dried (yield = 4.1 mg, 9.8%). The phase purity of this compound is confirmed by powder X-ray diffraction analysis (Figure S2).

### Synthesis of [Co­(NA)­(H_2_O)_2_] (**4**)

A clear yellow solution containing the salt Na_2_(NA)·2H_2_O (99.2 mg; 0.32 mmol) in 15 mL of H_2_O is added, dropwise, over a pink solution containing Co­(CH_3_COO)_2_·4H_2_O (39.8 mg; 0.16 mmol), LiCl (6.7 mg; 0.16 mmol) and NH_4_Cl (8.5 mg; 0.16 mmol) in 10 mL of H_2_O under stirring. The final solution is stirred for 20 min and left to evaporate at room temperature covered by parafilm with a few small pinholes. After 5 weeks, red plate-like crystals, suitable for single crystal X-ray diffraction, were obtained, filtered and air-dried (yield = 14.3 mg, 27.7 %). The phase purity of this compound is confirmed by powder X-ray diffraction analysis (Figure S3).

### Physical Measurements

Magnetic susceptibility of polycrystalline samples of compounds **2–4** was measured on a Quantum Design MPMS-XL-5 SQUID susceptometer in the temperature range 2–300 K with an applied magnetic field of 0.1 T. The susceptibility data were corrected for the sample holder and the corresponding diamagnetic contribution was evaluated using Pascal’s constants.^29^

### Crystallographic Data Collection and Refinement

Single crystals of compounds **1–4** were mounted on a MITIGEN loop using Paratone oil to coat the crystal and then transferred directly to a cold nitrogen stream. Data were collected using a XtaLAB Synergy R, DW system, HyPix diffractometer operating at 120 K. Data were measured using ω scans with Cu Kα radiation (λ = 1.54184 Å). The structures were solved with the ShelXT 2018/2 ^30^ solution program using dual methods and Olex2 1.5 as the graphical interface.^31^ The model is refined with version 2017/1 of XL^32,33^ using the least-squares minimization. All non-hydrogen atoms were refined anisotropically. Most hydrogen atom positions were calculated geometrically and refined using the riding model, but some hydrogen atoms were refined freely. Compound **1** crystallizes in the monoclinic *C*2/*c* space group, compounds **2** and **3** in the triclinic *P*-1 space group and compound **4** in the monoclinic *I*2*/a* space group (Table 4).

**Table 4:** Crystal Data and Structure Refinement of Compounds (PPh_4_)_4_[Co­(NA)_3_] (**1**), (NMe_4_)_2_[Co_2_(NA)_3_(H_2_O)_4_] (**2**), (NPr_4_)_2_[Co_3_(NA)_4_(H_2_O)_6_] (**3**) and [Co­(NA)­(H_2_O)_2_] (**4**)

compound	**1**	**2**	**3**	**4**
formula	C_114_H_80_CoN_6_O_24_P_4_	C_26_H_32_Co_2_N_10_O_28_	C_48_H_68_Co_3_N_10_O_38_	C_6_H_4_CoN_2_O_10_
CCDC	2424896	2424897	2424898	2424899
*D*_calc._/g cm^–3^	1.438	1.889	1.656	2.308
μ/mm^–1^	2.703	8.166	7.104	15.201
Formula Weight	2100.65	1050.47	1569.91	323.04
Color	orange	orange	orange	red
Shape	prism	hexagonal	plate-shaped	rhombohedral
Size/mm^3^	0.11 × 0.04 × 0.01	0.06 × 0.03 × 0.01	0.07 × 0.05 × 0.01	0.08 × 0.06 × 0.02
*T*/K	120.0(3)	120.0(3)	120.0(3)	120.0(3)
Crystal System	monoclinic	triclinic	triclinic	monoclinic
Space Group	*C*2/*c*	*P*-1	*P*-1	*I*2/*a*
a/Å	25.8417(3)	9.1388(4)	9.2796(3)	9.6819(2)
b/Å	13.6597(2)	9.2508(4)	11.9825(4)	6.72530(10)
c/Å	27.8431(4)	11.7835(4)	15.18040(10)	14.4245(3)
α/°	90	101.708(3)	97.838(2)	90
β/°	99.1940(10)	106.742(4)	93.475(2)	98.200(2)
γ/°	90	94.777(4)	108.675(3)	90
V/Å^3^	9702.1(2)	923.38(7)	1574.33(8)	929.63(3)
*Z*	4	1	1	4
Wavelength/Å	1.54184	1.54184	1.54184	1.54184
Radiation type	Cu K_*a*_	Cu K_*a*_	Cu K_*a*_	Cu K_*a*_
Θ_*min*_/°	3.216	4.033	2.957	6.200
Θ_max_/°	76.462	67.010	67.030	66.602
measured Refl’s	45769	15600	38704	4225
indep’t Refl’s	9780	3259	5578	819
Refl’s I ≥ 2 σ(I)	8283	2894	4856	791
*R*_int_	0.0451	0.0693	0.0819	0.0228
parameters	672	304	467	90
restraints	135	64	46	16
largest peak	1.298	1.653	1.145	1.003
deepest hole	–1.065	–1.395	–0.873	–0.588
GooF	1.091	1.078	1.107	1.119
w*R*_2_ (all data)	0.1876	0.2669	0.1933	0.1299
w*R*_2_	0.1717	0.2575	0.1869	0.1174
*R*_1_ (all data)	0.0782	0.0927	0.0699	0.0464
*R*_1_	0.0666	0.0860	0.0640	0.0437

A summary of the data collection and structure refinements for compounds **1–4** is given in Table 4. CCDC 2424896-9 contain the supplementary crystallographic data for compounds **1–4**, respectively. Tables S1–S8 in the Supporting Information contain the bond distances and angles for compounds **1–4**.

## Results and Discussion

### Syntheses of the Compounds

Compounds **1–4** have been prepared by slightly changing the synthetic conditions. The main change is the salt used to precipitate the cobalt/nitranilato anion formed: PPh_4_Br for **1**, NMe_4_Br for **2**, NPr_4_Br for **3** and NH_4_Cl for **4** (although in **4** we obtain a neutral chain, see below). In all cases, the synthesis is performed by reaction of a cobalt­(II) salt (chloride for **1** and **3** and acetate for **2** and **4**) with the sodium salt of the nitranilato ligand in the presence of LiCl and the corresponding cation (PPh_4_^+^, NMe_4_^+^, NPr_4_^+^ and NH_4_^+^ for **1–4**, respectively). The use of cobalt­(II) acetate for **1** and **3** or cobalt­(II) chloride for **2** and **4** resulted in lower quality single crystals. In the case of compound **3**, which seems to be the most difficult to crystallize (probably due to the larger size of the anion) we had to use a slow diffusion method to obtain high quality single crystals. Although the Li^+^ cations do not enter in the crystal structure, their presence is needed to obtain good quality single crystals in all cases. We suppose that they play a template role favoring an initial close packing of the anions.

The synthesis of compounds **1–4** shows the key role of the size of the cation in stabilizing and crystallizing the different anions. Interestingly, the smallest cation NH_4_^+^ cannot stabilize any anion and, therefore, it does not enter the structure, leading to the formation of the neutral chain [Co­(NA)­(H_2_O)_2_] (**4**). Note that compound **4** has also been prepared by layering an aqueous solution of Na_2_(NA)·2H_2_O with an ethanolic solution of Co­(NO_3_)_2_·6H_2_O without any extra cation. Finally, it is worth noting that when using other cations such as NEt_4_^+^ we obtain the chain compound **4** and with NBu_4_^+^, no single crystals nor powder were obtained.

### Description of the Structures

#### Structure of (PPh_4_)_4_[Co­(NA)_3_] (**1**)

Compound **1** crystallizes in the monoclinic *C*2/*c* space group (Table 4). Its asymmetric unit contains one cobalt­(II) atom, located on a 2-fold axis, one and a half coordinated nitranilato ligands and two complete PPh_4_^+^ cations (noted as A and B), located on general positions (Figure S4). Application of the 2-fold axis generates the formula (PPh_4_)_4_[Co­(NA)_3_]. The structure of **1** shows the presence of two different, slightly interpenetrated, layers parallel to the *ab* plane (Figure 1a). One of the layers contains the A-type PPh_4_^+^ cations (centered on the P1A atom, Figure 1b) and the [Co­(NA)_3_]^4–^ anions (Figure 1c). The other layer contains only the B-type PPh_4_^+^ cations (centered on the P1B atom, Figure 1d). There are two different anionic layers in the unit cell. One contains the Δ *e*nantiomers of the chiral [Co­(NA)_3_]^4–^ anions and the other the Λ ones. The chiral [Co­(NA)_3_]^4–^ anion is stabilized by the formation of up to six π–π interactions between the three anilato rings and the aromatic phenyl groups of the surrounding PPh_4_^+^ cations, with centroid–centroid distances of 3.5334(6), 4.0261(5) and 4.1819(3) Å (there are two of each type, Figure 2). These six π–π interactions were also key in the stabilization of the closely related chiral anions [Fe­(C_6_O_4_X_2_)_3_]^3–^ (X = Cl and Br) with the PPh_3_Et^+^ cation.^19^

**Figure 1:**
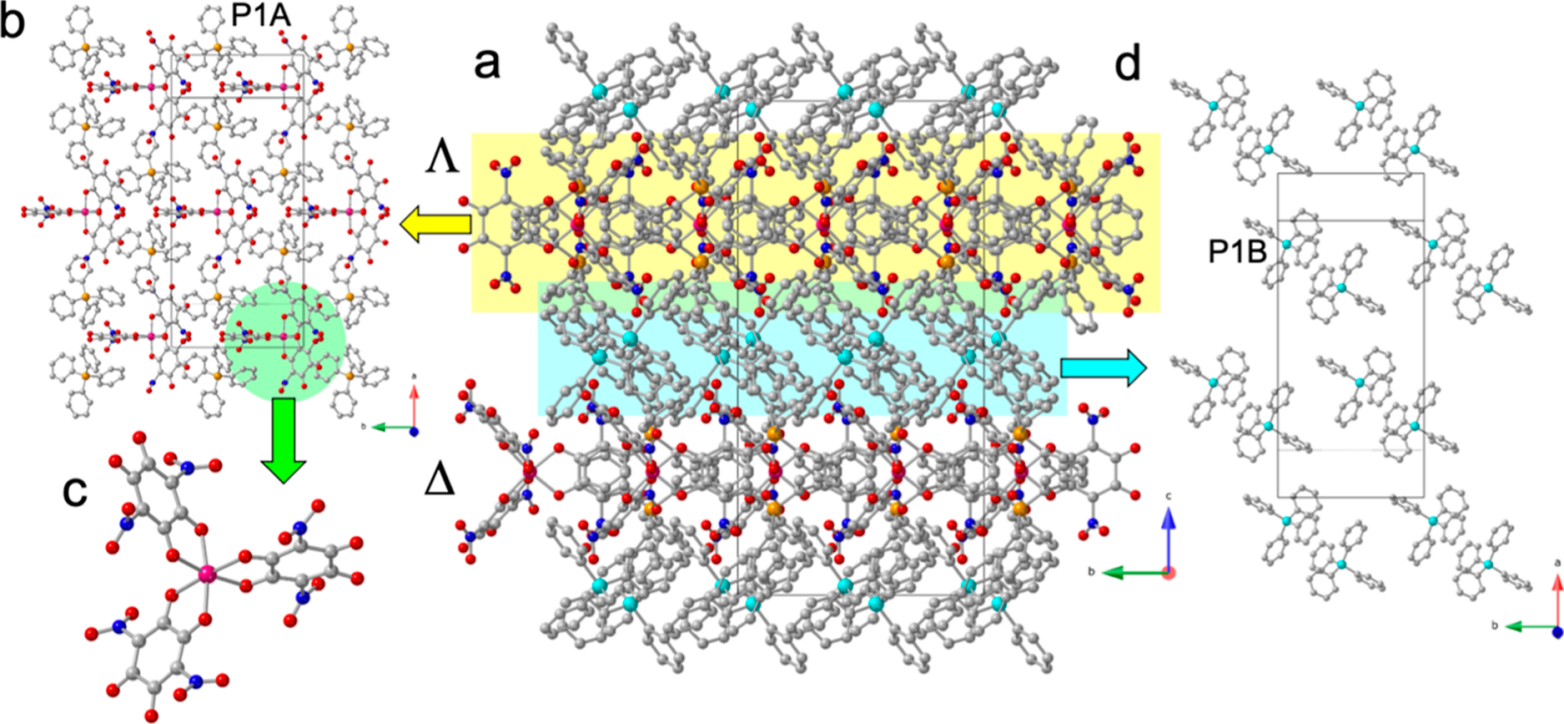
Structure of compound (PPh_4_)_4_[Co­(NA)_3_] (**1**). (**a**) View of the alternating layers parallel to the ab plane. (**b**) View, along the *c* direction, of the layer containing the anions and A-type PPh_4_^+^ cations. (**c**) View of the [Co­(NA)_3_]^4–^ anion. (**d**) View, along the *c* direction, of the layer containing B-type PPh_4_^+^ cations. Color code: Co = pink, C = gray, O = red, N = dark blue, P1A = orange and P1B = cyan. H atoms are omitted for clarity.

**Figure 2:**
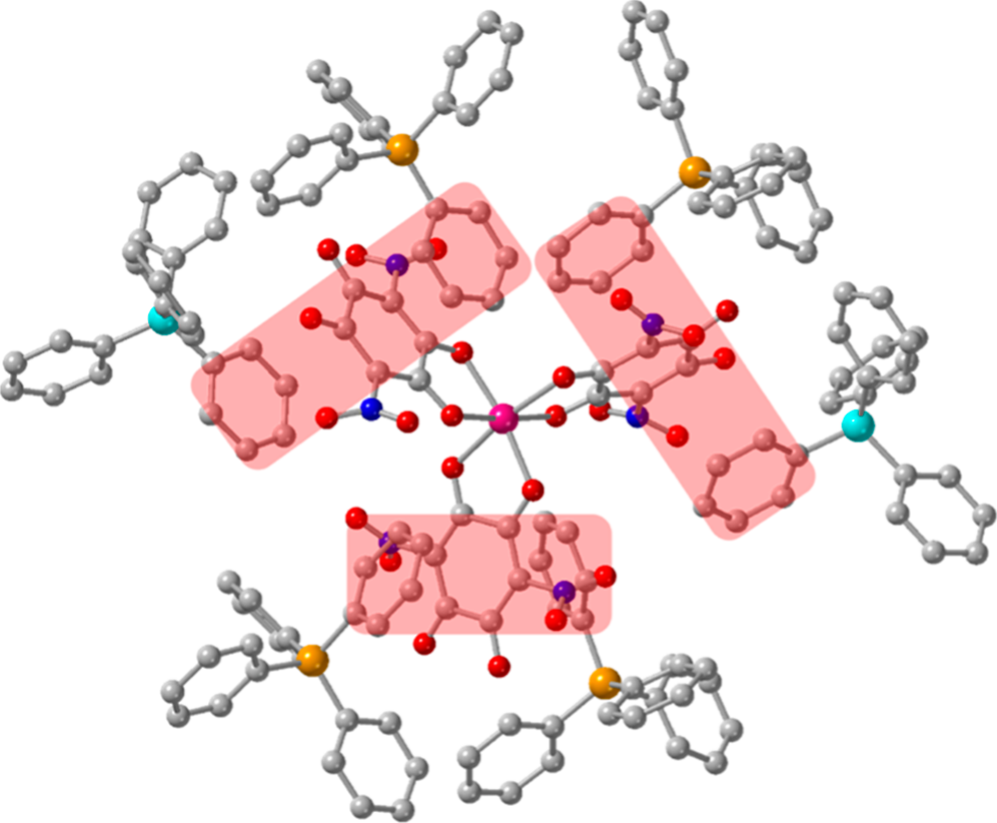
View of the [Co­(NA)_3_]^4–^ anion surrounded by six PPh_4_^+^ cations showing the six π–π interactions (red rectangles). Color code: Co = pink, C = gray, O = red, N = dark blue, P1A = orange and P1B = cyan. H atoms are omitted for clarity.

The [Co­(NA)_3_]^4–^ anion is formed by a central cobalt­(II) atom surrounded by three bidentate terminal nitranilato ligands in a slightly distorted octahedral geometry (Figure 1c), as observed in all the [M­(C_6_O_4_X_2_)_3_]^3–^ anions prepared to date. The analysis of the coordination environment of the Co^II^ ion in **1** with the program SHAPE^34,35^ (Table S9) shows that it presents a slightly distorted octahedral coordination geometry (Figure 1c).

Interestingly, a search in the CCDC database shows a total of 34 anilato-based compounds containing [M^III^(C_6_O_4_X_2_)_3_]^3–^ trianions with M^III^ = Fe (26 examples), Cr^III^ (6 examples), Ga^III^ and In^III^ (one example each). Surprisingly, no example with Co^III^ or with any divalent metal ion is reported. Therefore, the [Co­(NA)_3_]^4–^ anion in **1** is the first example of tris­(anilato)­cobaltate anion and also the first tris­(anilato)­metalate with any divalent metal ion. The Co–O bond distances in **1** (in the range 2.076–2.093 Å, with an average value of 2.085 Å, Table S1) are similar to those observed in the only two reported related tris­(oxalate)­cobaltate­(II) anions: [Co^II^(C_2_O_4_)_3_]^4–^ (that show average values of 2.089 and 2.090 Å).^36,37^ As expected, these values are longer than those observed in the related [Co^III^(C_2_O_4_)_3_]^3–^ anions (where the average values are around 1.890–1.910 Å).^38–42^ Furthermore, BVS calculations clearly show that the oxidation state of the cobalt atom in compound **1** is +2 (as in compounds **2–4**, Table S10).^43^

#### Structure of (NMe_4_)_2_[Co_2_(NA)_3_(H_2_O)_4_] (**2**)

Compound **2** crystallizes in the triclinic *P*-1 space group (Table 4). Its asymmetric unit contains one cobalt­(II) atom, one and a half nitranilato ligands, two coordinated water molecules and a NMe_4_^+^ cation, located on general positions (Figure S5). Application of the inversion center (located in the center of the half nitranilato) generates the formula (NMe_4_)_2_[Co_2_(NA)_3_(H_2_O)_4_] (**2**). The structure of **2** consists of anionic layers, parallel to the *ac* plane, containing [Co_2_(NA)_3_(H_2_O)_4_]^2–^ dimers alternating with cationic layers containing the NMe_4_^+^ cations (Figure 3a). The anionic layers are formed by discrete [Co_2_(NA)_3_(H_2_O)_4_]^2–^ dimers that are H-bonded (with eight H-bonds of types b–e, Figure 4) forming ribbons running along the *a* direction. These ribbons are further H-bonded to neighboring ribbons (with two H-bonds of type-a, Figure 4), generating a H-bonded layer (Figures 3b and 4). Table S11 shows the structural parameters of all these H-bonds.

**Figure 3:**
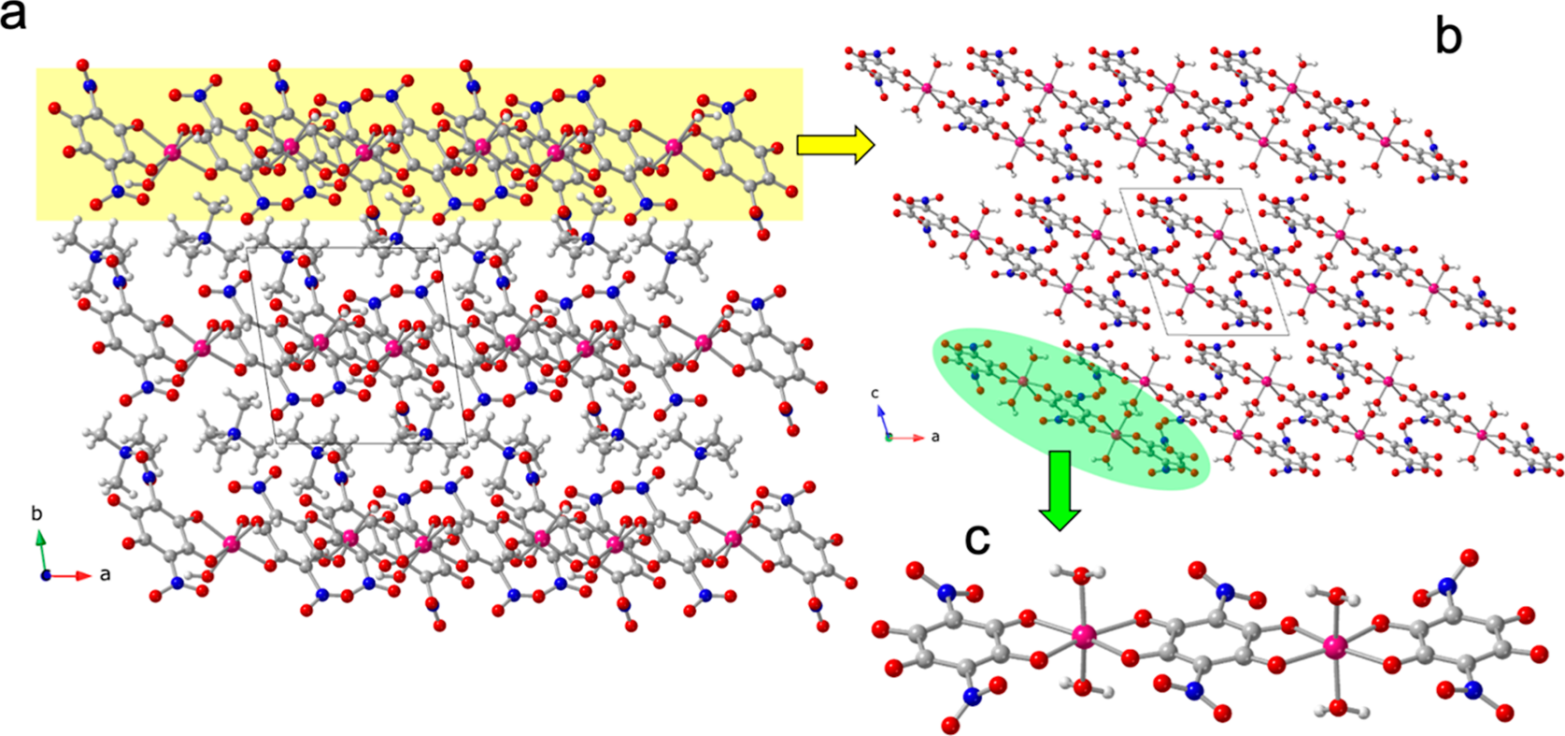
Structure of compound (NMe_4_)_2_[Co_2_(NA)_3_(H_2_O)_4_] (**2**). (a) View of the alternating layers parallel to the ac plane. (b) View, down the *b* direction, of the anionic layer. (c) View of the [Co_2_(NA)_3_(H_2_O)_4_]^2–^ anion. Color code: Co = pink, C = gray, O = red, N = dark blue and H = white.

**Figure 4:**
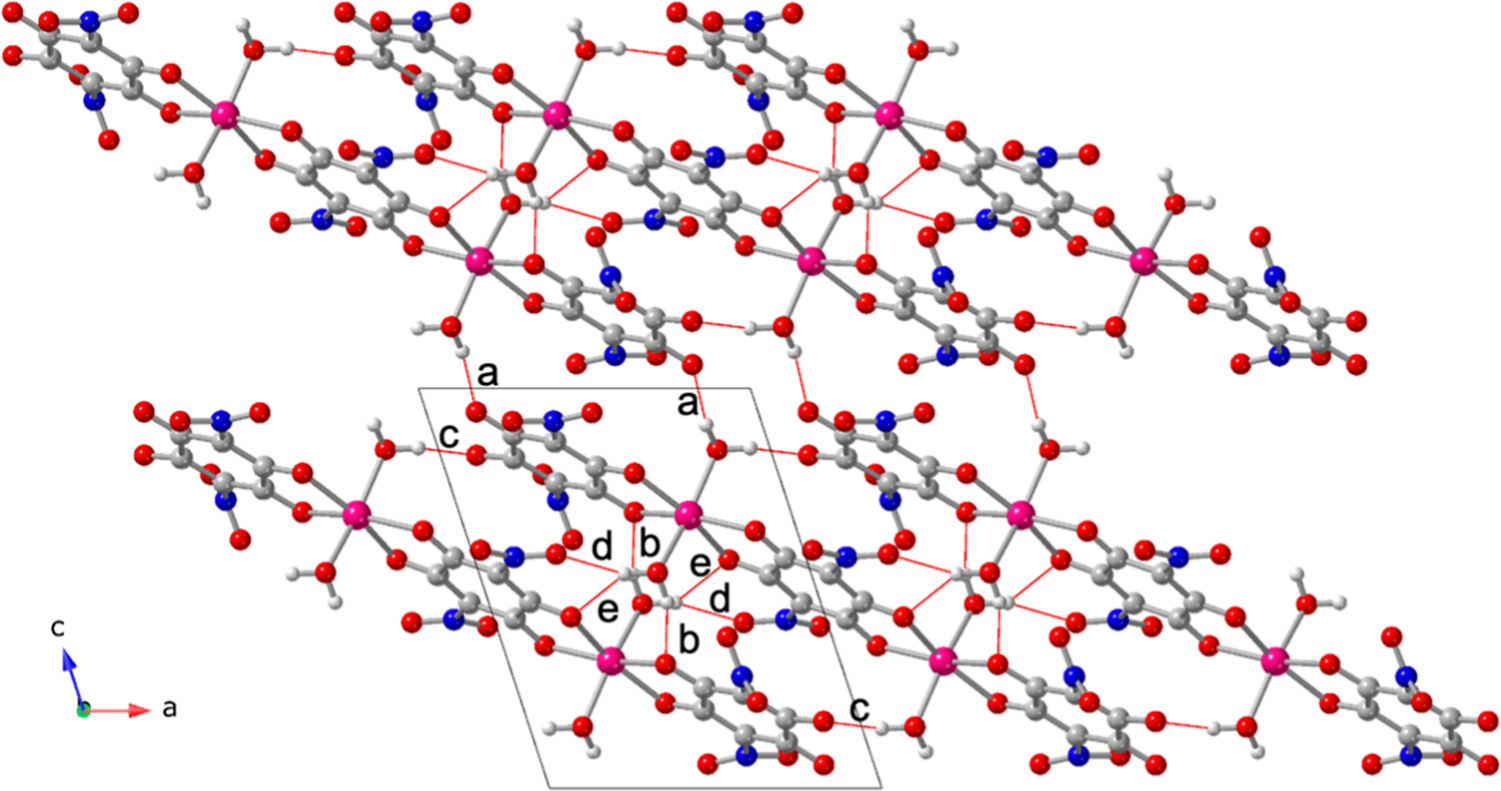
Anionic layer in compound (NMe_4_)_2_[Co_2_(NA)_3_(H_2_O)_4_] (**2**) showing the interdimer H-bonds as thin red lines. Letters a-e indicate the H-bond type (see Table S11). Color code: Co = pink, C = gray, O = red, N = dark blue and H = white.

The centrosymmetric [Co_2_(NA)_3_(H_2_O)_4_]^2–^ dimers contain two cobalt­(II) atoms connected by a central bis-bidentate bridging nitranilato ligand (Figure 3c). The Co^II^ centers complete their octahedral coordination geometry with a terminal coordinated chelating nitranilato ligand and two trans water molecules. The Co–O bond distances (in the range 2.034–2.146 Å with an average value of 2.087 Å, Table S3) are similar to those found in compound **1** suggesting that the oxidation state of the cobalt centers is +2, in agreement with the presence of two NMe_4_^+^ cations per dimer. Furthermore, as in **1**, the BVS calculations clearly show that the oxidation state of the cobalt atom in compound **2** is also +2 (Table S10).^43^ The analysis of the coordination environment of the Co^II^ ion in **2** with the program SHAPE^34,35^ (Table S9) shows that it presents a slightly distorted octahedral coordination geometry.

#### Structure of (NPr_4_)_2_[Co_3_(NA)_4_(H_2_O)_6_] (**3**)

Compound **3** crystallizes in the triclinic *P*-1 space group (Table 4). Its asymmetric unit contains two cobalt­(II) atoms (Co1 and Co2), two nitranilato ligands, three coordinated water molecules and a NPr_4_^+^ cation, all located on general positions except the Co2 center, which is located on an inversion center (Figure S6). Application of the inversion center generates the formula (NPr_4_)_2_[Co_3_(NA)_4_(H_2_O)_6_] (**3**). The structure of **3** is similar to that of compound **2**: it consists of anionic layers, parallel to the *ac* plane, containing [Co_3_(NA)_4_(H_2_O)_6_]^2–^ trimers alternating with cationic layers containing the NPr_4_^+^ cations (Figure 5a). The anionic layers are formed by discrete [Co_3_(NA)_4_(H_2_O)_6_]^2–^ trimers that are H-bonded (with six H-bonds of types b-d, Figure 6) forming ribbons running along the *a* direction. These ribbons are further H-bonded to neighboring ribbons (with two H-bonds of type a, Figure 6), generating a H-bonded layer (Figures 5b and 6). Table S12 shows the structural parameters of all these H-bonds.

**Figure 5:**
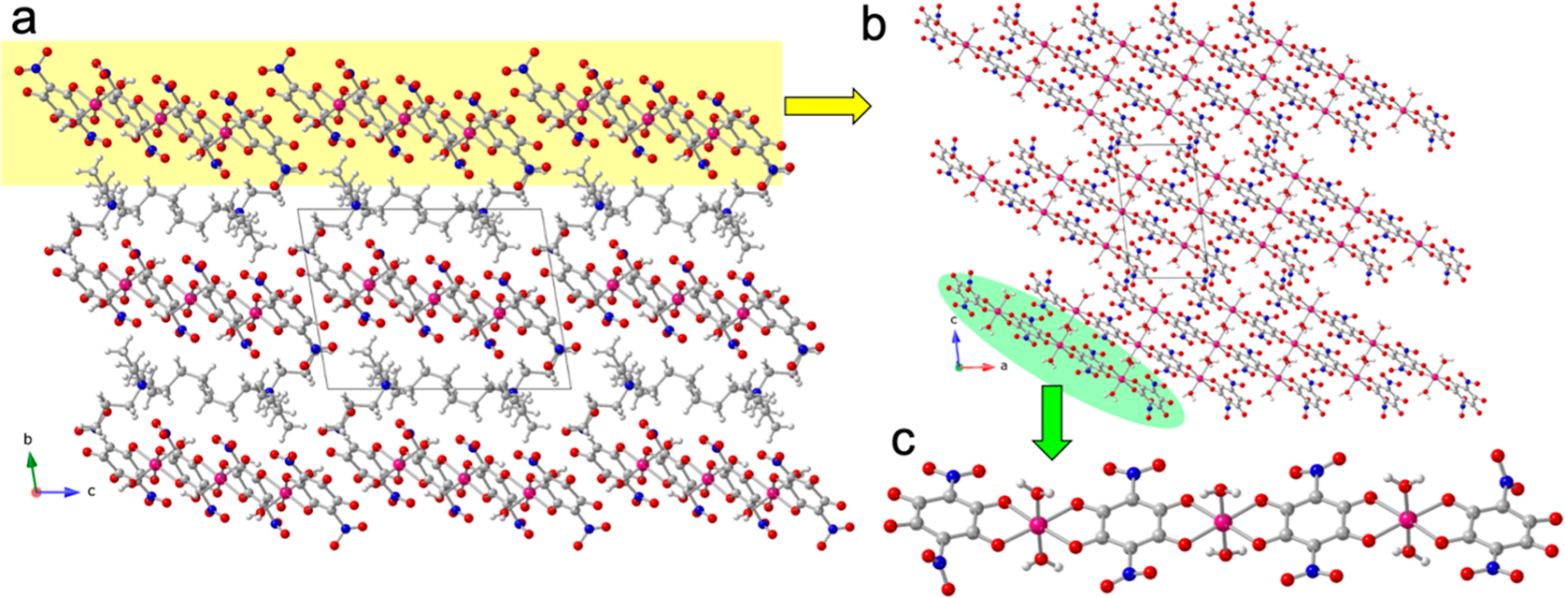
Structure of compound (NPr_4_)_2_[Co_3_(NA)_4_(H_2_O)_6_] (**3**). (a) View of the alternating layers parallel to the ac plane. (b) View, down the *b* direction, of the anionic layer. (c) View of the [Co_3_(NA)_4_(H_2_O)_6_]^2–^ anion. Color code: Co = pink, C = gray, O = red, N = dark blue and H = white.

**Figure 6:**
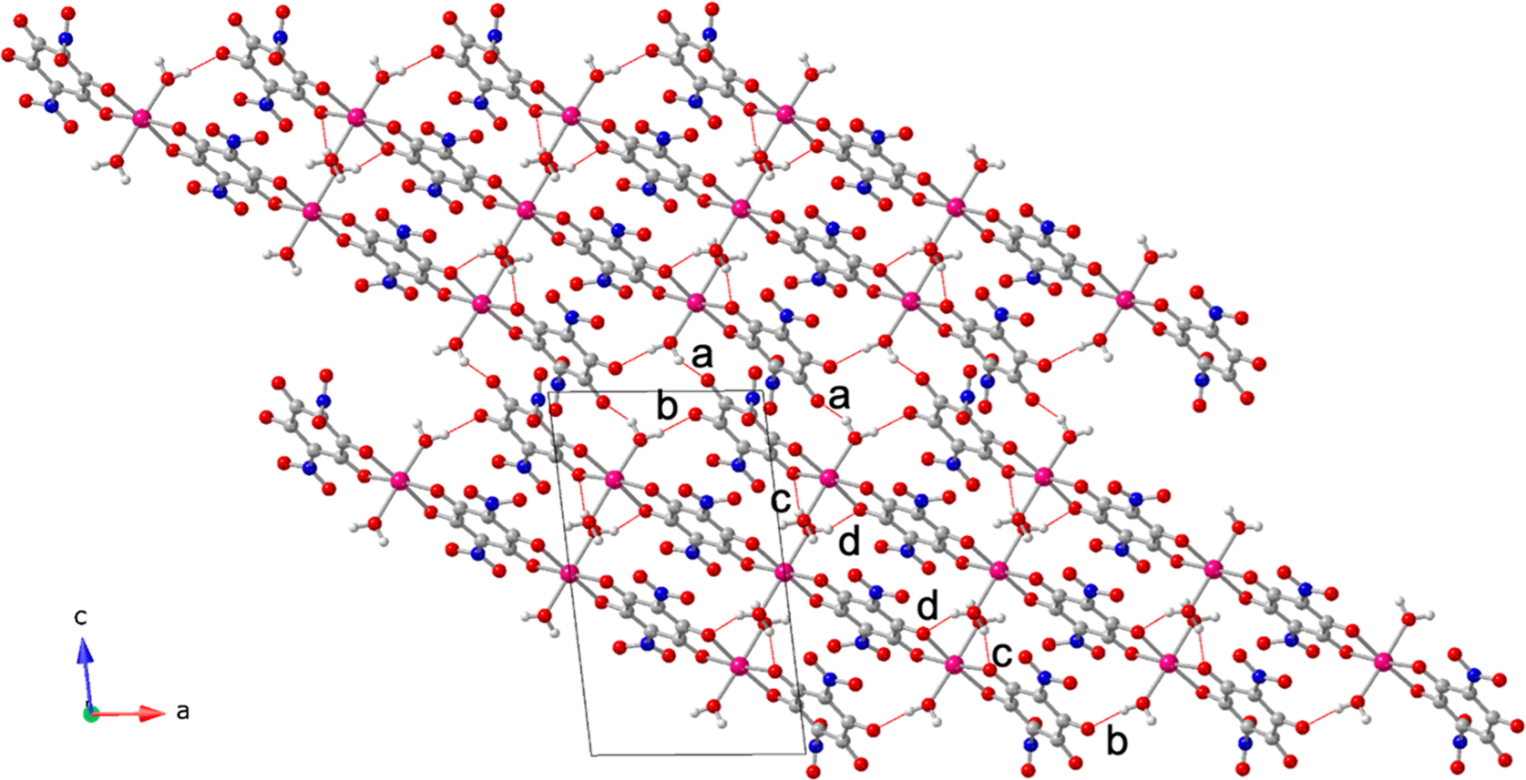
Anionic layer in compound (NPr_4_)_2_[Co_3_(NA)_4_(H_2_O)_6_] (**3**) showing the intertrimer H-bonds as thin red lines. Letters a-d indicate the H-bond type (see Table S12). Color code: Co = pink, C = gray, O = red, N = dark blue and H = white.

The centrosymmetric [Co_3_(NA)_4_(H_2_O)_6_]^2–^ trimers contain a central cobalt­(II) atom connected to the two external cobalt­(II) atoms by two bis-bidentate bridging nitranilato ligands (Figure 5c). The central cobalt­(II) center completes its octahedral coordination geometry with two trans water molecules, whereas the external cobalt­(II) centers complete their octahedral coordination geometry with a terminal coordinated chelating nitranilato ligand and two trans water molecules. The Co–O bond distances (in the range 2.044–2.137 Å for Co1 and 2.044–2.102 Å for Co2, with average values of 2.083 Å for Co1 and 2.074 Å for Co2, Table S5) are similar to those found in compounds **1** and **2**, suggesting that in **3** the oxidation state of the cobalt centers is also +2, in agreement with the presence of two NPr_4_^+^ cations per trimer. Furthermore, as in **1** and **2**, the BVS calculations clearly show that the oxidation state of both cobalt atoms in compound **3** is also +2 (Table S10).^43^ Finally, the analysis of the coordination environment of both Co^II^ ions with the program SHAPE^34,35^ (Table S9) shows that they present a slightly distorted octahedral coordination geometry.

#### Structure of Co­[(NA)­(H_2_O)_2_] (**4**)

The structure of this compound has already been reported (although at 293 K, CCDC code ROJLAG)^13^ and, therefore, here we will only make a brief description of its structure and compare it with that of compounds **1–3**. The asymmetric unit of compound **4** contains a cobalt­(II) center, located on a 2-fold axis, half nitranilato ligand and a coordinated water molecule (Figure S7). Application of the 2-fold axis generates neutral zigzag chains [Co­(NA)­(H_2_O)_2_]_*n*_, running along the *c* axis, where the Co^II^ ions are connected through bridging bis-bidentate nitranilato ligands with two cis water molecules completing their slightly distorted octahedral geometry (Figure 7). The Co–O bond distances (in the range 2.064–2.115 Å, with an average value of 2.083 Å, Table S7) are similar to those found in compounds **1–3**, suggesting a charge of +2 for the Co center, in agreement with stoichiometry. BVS calculations also confirm this +2 oxidation state, as in 1–**3** (Table S10).^43^ The coordination geometry is slightly distorted octahedra, as shown by the continuous Shape analysis (Table S9).^34,35^

**Figure 7:**
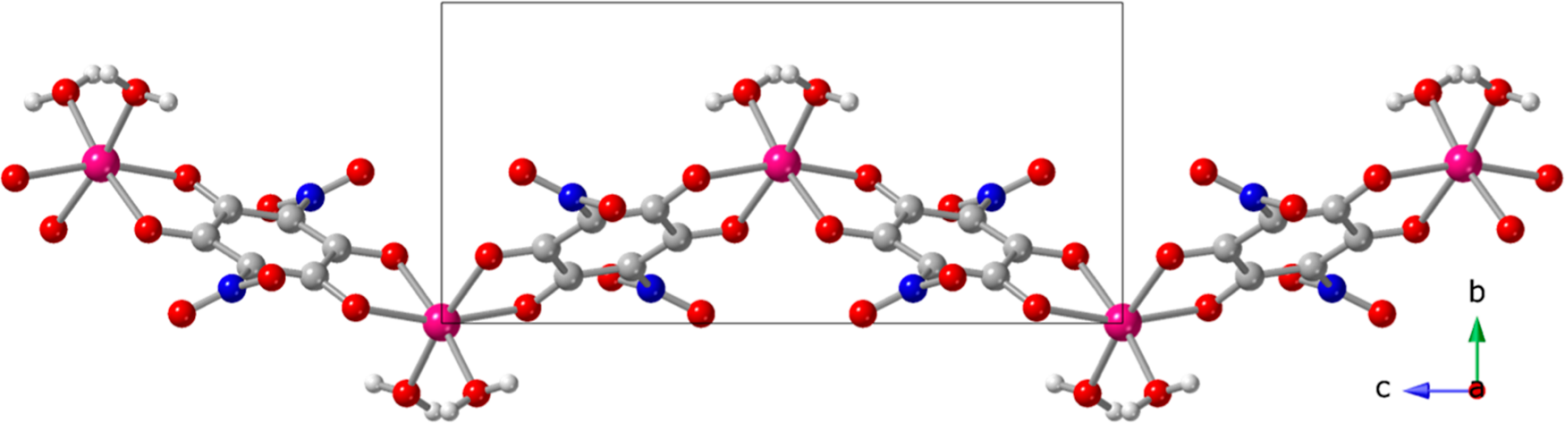
Structure of compound [Co­(NA)­(H_2_O)_2_] (**4**). Color code: Co = pink, C = gray, O = red, N = dark blue and H = white.

### Role of the Cations on the Structures of Compounds **1–4**

The structures of compounds **1–4** show some interesting facts in the cobalt­(II)/nitranilato system: (i) There are, at least, three possible anions and one neutral chain that can be formed in aqueous solution. (ii) These four compounds show different nitranilato/cobalt­(II) ratios (3:1 in **1**, 3:2 in **2**, 4:3 in **3** and 1:1 in **4**), which are different to the used in the synthesis: (1:5 in **1**, 3:1.6 in **2**, 3:2 in **3** and 2:1 in **4**). (iii) The four compounds also show different ratios between terminal and bridging nitranilato ligands (3:0 in **1**, 2:1 in **2**, 2:2 in **3** and 0:1 in **4**, Figure 8). (iv) The cations used to crystallize compounds **1**–**4** are also different: (PPh_4_^+^ in **1**, NMe_4_^+^ in **2**, NPr_4_^+^ in **3** and NH_4_^+^ in **4**).

**Figure 8:**
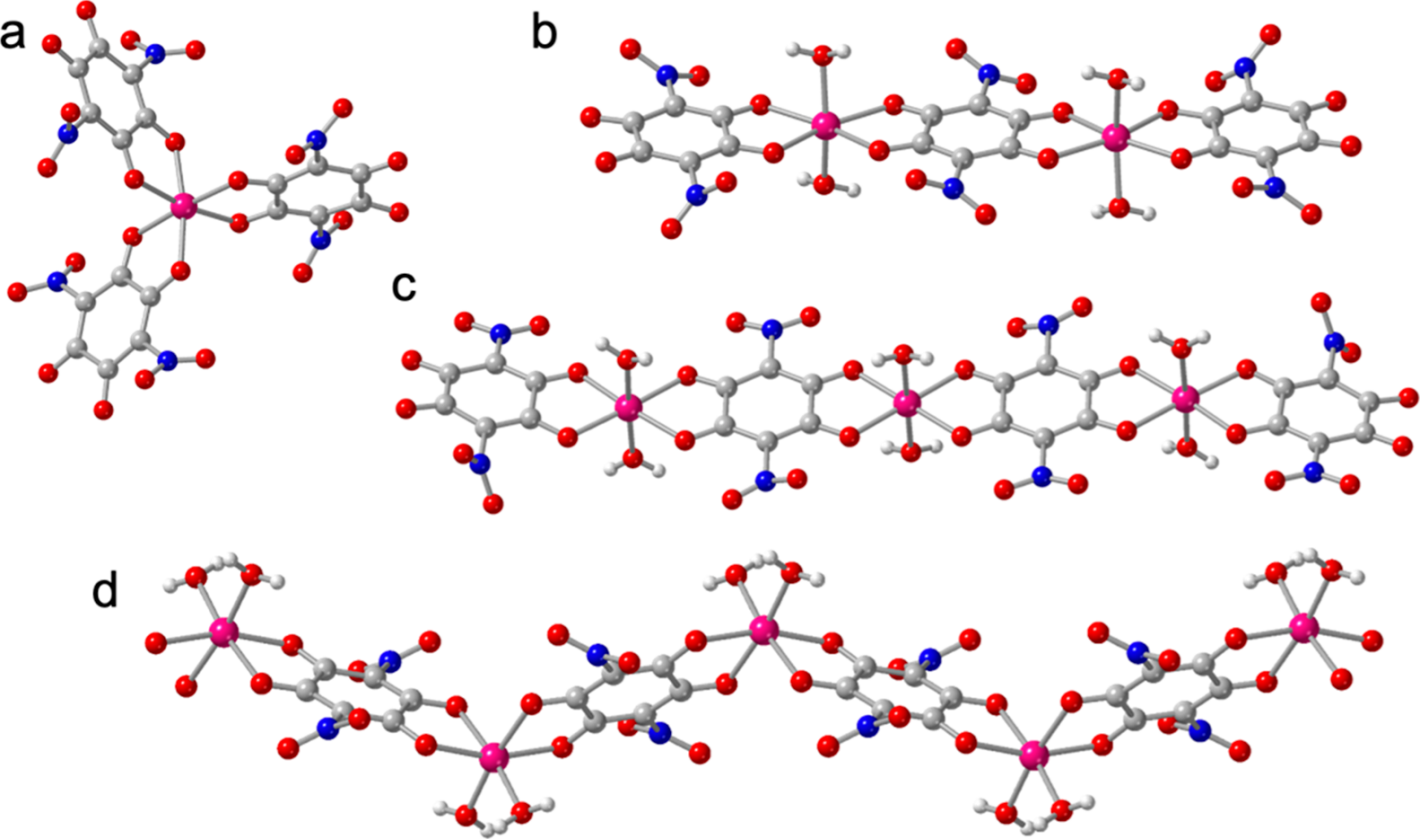
View of the structure of: (a) the [Co­(NA)_3_]^4–^ monomer in **1**, (b) the [Co_2_(NA)_3_(H_2_O)_4_]^2–^ dimer in **2**, (b) the [Co_3_(NA)_4_(H_2_O)_6_]^2–^ trimer in **3** and (d) the [Co­(NA)­(H_2_O)_2_] chain in **4**. Color code: Co = pink, C = gray, O = red, N = dark blue and H = white.

Since the observed ligand/metal ratios are different to the ones used in the synthesis, we can conclude that the main factor determining the final compound obtained is the cation used to crystallize it. This assumption is supported by the fact that compound **1** presents the highest nitranilato/cobalt ratio (3:1) despite we used the lowest ratio in the synthesis (1:5). In this case it is straightforward to realize that, besides the anion–cation interactions, we have up to six π–π interactions formed between the anilato rings of the [Co­(NA)_3_]^4–^ anion and the aromatic phenyl rings of six surrounding PPh_4_^+^ cations. In fact, these interactions are identical to those responsible of the stabilization of enantiopure crystals of compounds (PPh_3_Et)_3_[Fe­(C_6_O_4_X_2_)_3_]^3–^ (X = Cl and Br).^19^ In compounds **2** and **3**, the experimental nitranilato/cobalt ratios (3:1.6 and 3:2, respectively) are also different to those observed in the respective compounds (3:2 and 4:3). This fact suggests again the idea that the cation determines the final compound crystallized. Thus, as expected, in compound **2** the smaller NMe_4_^+^ cation crystallizes with the smaller anion (the [Co_2_(NA)_3_(H_2_O)_4_]^2–^ dimer) whereas in **3**, the larger NPr_4_^+^ cation crystallizes with the larger anion (the [Co_3_(NA)_4_(H_2_O)_6_]^2–^ trimer). The fact that the intermediate NEt_4_^+^ and larger NBu_4_^+^ cations do not crystallize with none of them, suggests that the final anion crystallized is very sensitive to the size of the cation. Finally, compound **4** further confirms this idea. In this case, the NH_4_^+^ cation is too small to crystallize with any anion (monomer, dimer or trimer) and, accordingly, the obtained compound is a neutral chain [Co­(NA)­(H_2_O)_2_] that does not requires any cation. In fact, the same chain has been synthesized by Molčanov and Milašinović without using any countercation.^13^ Finally, it is worth to mention that such structural diversity has not been observed with any other anilato ligand nor any other metal ion (although our preliminary results indicate that Fe^II^ may show a similar behavior to Co^II^). This uniqueness of nitranilato may be attributed to the fact that the other anilato ligands show almost exclusively the bis-bidentate bridging coordination mode when combined with divalent transition metals, leading to coordination polymers (either 1D, 2D or 3D), but not to discrete anions. A possible reason may be large electron-withdrawing effect of the NO_2_ group (that delocalizes the electron density in the two N–O bonds) reducing the electron density on the anilato ring, and, therefore, on the terminal oxygen atoms. This fact limits the coordination ability of the nitranilato ligand, resulting in a higher probability to act as bidentate (terminal) rather than bis-bidentate (bridging).

### Magnetic Properties

Since we only obtained a few single crystals of compound **1** and we can assume that it must be well isolated from the magnetic point of view, we have only performed magnetic measurements of compounds **2–4**. These magnetic properties of compounds **2–4** are quite similar: the product of the molar magnetic susceptibility per formula unit times the temperature (χ_m_T) shows in compounds **2–4** room temperature values of 6.4, 8.6, and 3.1 cm^3^ K mol^–1^ (Figure 9), corresponding to *ca*. 3.2, 2.9, and 3.1 cm^3^ K mol^–1^ per Co^II^ center, respectively. These values are within the normal range observed for isolated or quasi-isolated high spin cobalt­(II) atoms with a ^4^T_1g_ ground state and an important first order orbital contribution.^44^ When the temperature is decreased, the χ_m_T product remains constant in the temperature range 300–100 K and shows a progressive decrease to reach values at 2 K of 1.1, 3.5, and 0.8 cm^3^ K mol^–1^ for **2–4**, respectively. This behavior suggests the presence of a very weak antiferromagnetic coupling between the Co^II^ centers connected through the nitranilato ligands, although the decrease may also be due to the presence of the spin–orbit coupling in the Co^II^ centers.^44^

**Figure 9:**
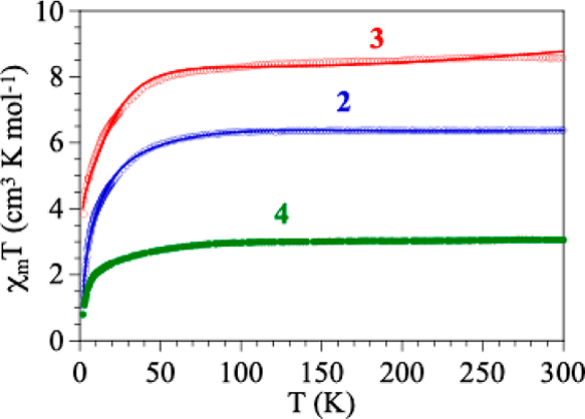
Thermal variation of the χ_m_T product for compounds **2–4**. Solid lines are the best fit to the models (see text).

In order to determine if there is a magnetic coupling, we have fitted the magnetic data of compounds **2** and **3** with the help of the program PHI.^45^ Since the structure of **2** shows a nitranilato-bridged symmetric Co^II^ dimer, we have fitted the magnetic properties of **2** with a Co^II^ dimer with a couplin constant (*J*), including a spin–orbit coupling (λ), an orbital reduction parameter (σ) and a zero-field splitting (ZFS) in both Co^II^ centers (D). This model reproduces very satisfactorily the magnetic data of compound **2** in the whole temperature range with the following parameters: *J* = −0.54(1) cm^–1^, σ = −1.27(1), λ = −34.0(3) cm^–1^, and |*D*| = 1.3(3) cm^–1^ (with a Hamiltonian of the type: *Ĥ* = −2J [*Ŝ*_1_ *Ŝ*_2_], solid line in Figure 9).

For compound **3**, since the structure shows a symmetric Co^II^ trimer with the same nitranilato bridges, we have fitted its magnetic properties with a Co^II^ trimer model with the following parameters: *J* = −0.08(1) cm^–1^, σ = −0.91(1), λ = −46.3(5) cm^–1^ and |*D*| = 2.3(1) cm^–1^ (with a Hamiltonian of the type *Ĥ* = −2J [*Ŝ*_1_ *Ŝ*_2_ + *Ŝ*_2_ *Ŝ*_3_], solid line in Figure 9).

The observed *J* values in both compounds are very weak and antiferromagentic, in agreement with the values found in other anilato-bridged cobalt­(II) complexes.^46–51^

## Conclusions

We have shown how by simply changing the countercation, it is possible to crystallize up to three different Co-nitranilato anions and even a neutral chain. Thus, the use of the phenyl-containing PPh_4_^+^ cation results in the crystallization of compound (PPh_4_)_4_[Co­(NA)_3_] (**1**), that contains the first reported tetra-anion of the type [M^II^(L)_3_]^4–^, with any M^II^ and anilato ligand. This hitherto unknown anion is stabilized by the formation of up to six π–π interactions between the aromatic rings of the cation and the anilato rings. The use of tetramethylammonium cation leads to compound (NMe_4_)_2_[Co_2_(NA)_3_(H_2_O)_4_] (**2**), which contains a dianionic nitranilato-bridged cobalt­(II) dimer. When using the larger tetrapropylammonium cation, we obtain compound (NPr_4_)_2_[Co_3_(NA)_4_(H_2_O)_6_] (**3**) that contains a larger anilato-bridged dianionic trimer. Finally, when using a smaller cation as ammonium, we obtain the neutral chain [Co­(NA)­(H_2_O)_2_] (**4**), probably because the cation is too small to stabilize any discrete anion. These four compounds show the important role of the size and shape of the cations in the final compound formed. Interestingly, although the small cation NMe_4_^+^ gives rise to an anionic Co^II^ dimer and the larger cation NPr_4_^+^ results in an anionic Co^II^ trimer, the intermediate NEt_4_^+^ does not “fit” with any of those anions giving rise, as the NH_4_^+^ cation, to a neutral chain where no cation in need.

To determine the exact influence of the size of the cation in stabilizing one or another anion, crystallization attempts are in progress using different cations (besides NBu_4_^+^) such as tetraalkylammonium cations with two different alkyl groups as NR_3_R′^+^ or NR_2_R′_2_^+^. Furthermore, preliminary results show the possibility of extending this study to other divalent transition metal ions. Thus, if we replace Co^II^ by Fe^II^ we can also obtain the monomer, formulated as (PPh_4_)_4_[Fe­(NA)_3_], similar to **1**, also using PPh_4_^+^ as countercation. Moreover, we can also extend these results to other similar anilato-type ligands. Thus, preliminary results show that the closely related asymmetric chloronitranilato ligand, (C_6_O_4_(NO_2_)­Cl)^2–^, gives rise to the trimer (NPr_4_)_2_[Co_3_(C_6_O_4_(NO_2_)­Cl)_4_(H_2_O)_6_], similar to **3**, also when using NPr_4_^+^ as countercation.

A final interesting aspect of compounds **1–3** is the possibility of using them as precursors for synthesizing heterometallic lattices using the complex-as-ligand strategy, as has already been used to prepare other heterometallic anilato-based lattices.^4,52–55^

As observed in other anilato-bridged cobalt­(II) derivatives, compounds **2–4** show a very weak antiferromagnetic coupling mediated through the electron-poor ring of the nitranilato bridge.

## Supplementary Material



## Notes

Figures with the experimental and simulated powder X-ray diffractograms of compounds **2–4** and with structural details of compounds **1–4**. Tables with bond distances and angles, continuous Shape parameters and BVS calculation for compounds **1–4**, and with the H-bond parameters for compounds **2–4** (PDF)
