# Supplementary material for: Cation-Induced Structural Diversity in the Cobalt(II)/Nitranilato System: A Monomer, a Dimer, a Trimer and a Chain
Source: Cryst Growth Des. 2025 Mar 24;25(7):2215–26. doi: 10.1021/acs.cgd.5c00210 (PMC12128170; doi:10.1021/acs.cgd.5c00210)
Supplement: Supplementary file 1 [file cg5c00210_si_001.pdf]

Supporting Information

Cation-induced structural diversity in the cobalt(II)/nitranilato system: a monomer, a dimer, a trimer and a chain

Cristina Pintado-Zaldo, Louise Bureller, Gonzalo de Joz-Latorre, Carlos J. Gómez-García* and Samia Benmansour*

Departamento de Química Inorgánica. Universidad de Valencia. Dr. Moliner 50, 46100. Burjasot (Valencia) Spain. E-mail: sam.ben@uv.es (S.B.); carlos.gomez@uv.es (C.J.G.-G.)

Powder X-ray diffractograms

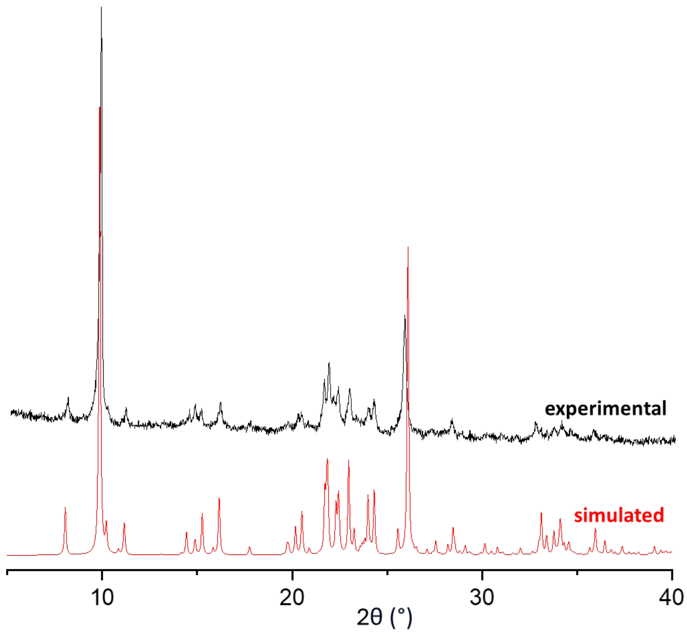

***Figure S1**. Experimental and simulated powder X-ray diffractograms of compound $(NMe_4)_2[Co_2(NA)_3(H_2O)_4]$ (**2**).*

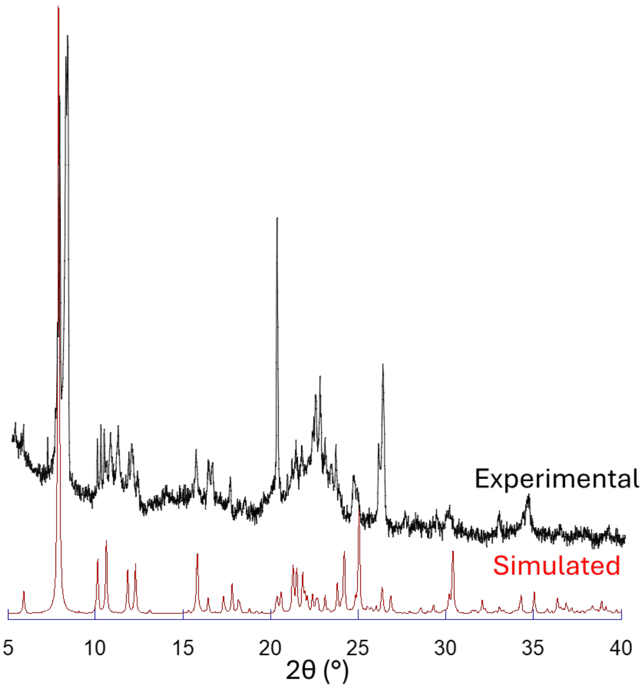

Figure S2. *Experimental and simulated powder X-ray diffractograms of compound* $(NPr_4)_2[Co_3(NA)_4(H_2O)_6]$ *(**3**).*

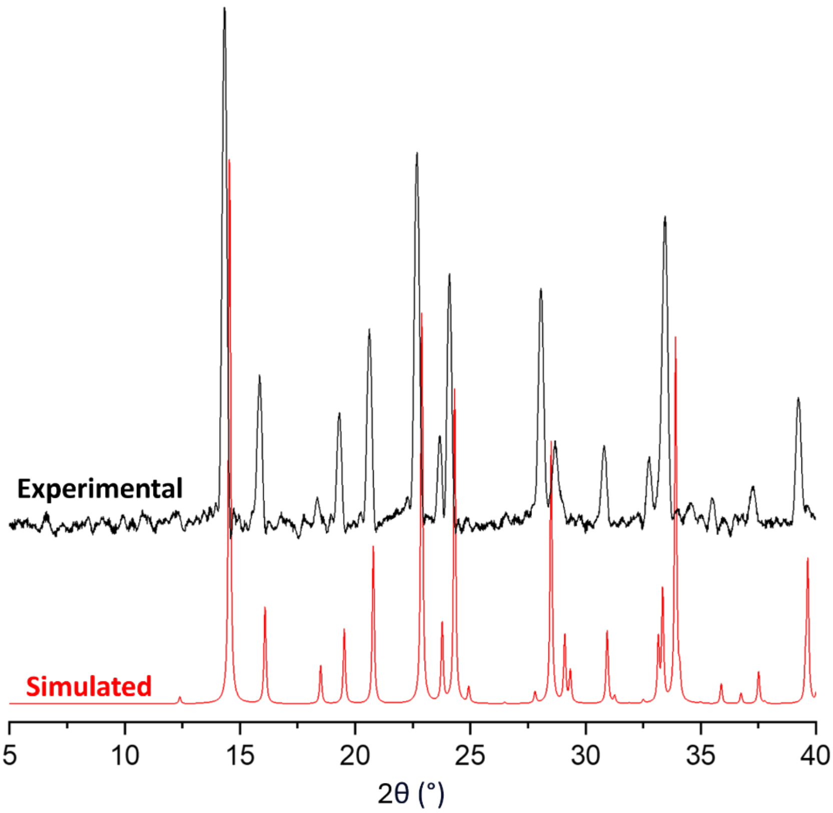

Figure S3. *Experimental and simulated powder X-ray diffractograms of compound* $[Co(NA)(H_2O)_2]$ *(**4**).*

Table S1. Bond distances (Å) in $(PPh_4)_4[Co(NA)_3]$ (**1**).

Atom	Atom	Length (Å)	Atom	Atom	Length (Å)
Co1	O3[1]	2.076(2)	C12	C11	1.398(5)
Co1	O3	2.076(2)	C8B	C9B	1.381(5)
Co1	O2	2.093(2)	C13B	C14B	1.389(5)
Co1	O2[1]	2.093(2)	C13B	C18B	1.394(5)
Co1	O12	2.087(2)	C1B	C2B	1.394(5)
Co1	O12[1]	2.087(2)	C1B	C6B	1.399(5)
P1A	C19A	1.795(3)	C14A	C15A	1.393(5)
P1A	C7A	1.802(3)	C11	C16	1.417(5)
P1A	C13A	1.794(3)	C20B	C21B	1.394(5)
P1A	C1A	1.796(3)	C24A	C23A	1.385(5)
P1B	C19B	1.799(3)	C4	C5	1.416(5)
P1B	C7B	1.788(3)	C1A	C6A	1.404(5)
P1B	C13B	1.791(3)	C1A	C2A	1.396(5)
P1B	C1B	1.790(3)	C16	C16[1]	1.559(7)
O3	C3	1.264(4)	C20A	C21A	1.392(5)
O2	C2	1.265(4)	C23A	C22A	1.376(5)
O12	C12	1.259(4)	C14B	C15B	1.391(5)
O16	C16	1.229(4)	C11B	C10B	1.387(5)
O4B	N4	1.121(4)	C18A	C17A	1.386(5)
N11	O11A	1.183(4)	C8A	C9A	1.386(5)
N11	O11B	1.214(4)	C2B	C3B	1.391(5)
N11	C11	1.487(4)	C9B	C10B	1.382(5)
O5	C5	1.239(5)	C12A	C11A	1.378(5)
N4	O4A	1.249(4)	C1	C6	1.434(5)
N4	C4	1.471(5)	C16A	C17A	1.382(6)
O6	C6	1.225(4)	C16A	C15A	1.373(6)
O1A	N1	1.151(5)	C6A	C5A	1.388(5)
N1	O2B	1.219(5)	C24B	C23B	1.380(5)
N1	C1	1.491(5)	C5	C6	1.530(5)
C3	C2	1.525(5)	C3B	C4B	1.380(6)
C3	C4	1.395(5)	C22A	C21A	1.384(6)
C19B	C20B	1.394(5)	C22B	C23B	1.386(5)
C19B	C24B	1.404(5)	C22B	C21B	1.387(5)
C19A	C24A	1.395(5)	C6B	C5B	1.381(6)
C19A	C20A	1.388(5)	C2A	C3A	1.391(6)
C2	C1	1.402(5)	C18B	C17B	1.383(6)
C12B	C7B	1.399(4)	C11A	C10A	1.378(6)
C12B	C11B	1.383(5)	C10A	C9A	1.373(6)
C7A	C8A	1.381(5)	C15B	C16B	1.383(7)
C7A	C12A	1.404(5)	C4B	C5B	1.377(7)
C13A	C14A	1.395(5)	C5A	C4A	1.388(7)
C13A	C18A	1.398(4)	C3A	C4A	1.386(7)

[1] 1-x, y, 3/2-z

Table S2. Bond angles (º) in $(PPh_4)_4[Co(NA)_3]$ (**1**).

Atom	Atom	Atom	Angle (˚)	Atom	Atom	Atom	Angle (˚)
O3^{1}	Co1	O3	92.53(12)	C14B	C13B	P1B	120.6(3)
O3^{1}	Co1	O2^{1}	77.82(9)	C14B	C13B	C18B	120.0(3)
O3	Co1	O2^{1}	99.06(9)	C18B	C13B	P1B	119.2(3)
O3	Co1	O2	77.83(9)	C2B	C1B	P1B	118.0(3)
O3^{1}	Co1	O2	99.07(9)	C2B	C1B	C6B	120.3(3)
O3^{1}	Co1	O12^{1}	170.10(9)	C6B	C1B	P1B	121.6(3)
O3	Co1	O12^{1}	94.79(9)	C15A	C14A	C13A	119.2(3)
O3	Co1	O12	170.10(9)	C12	C11	N11	118.0(3)
O3^{1}	Co1	O12	94.79(9)	C12	C11	C16	124.6(3)
O2	Co1	O2^{1}	175.58(14)	C16	C11	N11	117.4(3)
O12	Co1	O2	94.42(10)	C19B	C20B	C21B	118.9(3)
O12^{1}	Co1	O2	89.00(10)	C23A	C24A	C19A	119.4(3)
O12^{1}	Co1	O2^{1}	94.42(10)	C3	C4	N4	119.1(3)
O12	Co1	O2^{1}	89.00(10)	C3	C4	C5	124.1(3)
O12^{1}	Co1	O12	78.77(13)	C5	C4	N4	116.7(3)
C19A	P1A	C7A	105.99(15)	C6A	C1A	P1A	117.8(3)
C19A	P1A	C1A	110.51(15)	C2A	C1A	P1A	121.8(3)
C13A	P1A	C19A	108.79(15)	C2A	C1A	C6A	120.4(3)
C13A	P1A	C7A	113.16(15)	O16	C16	C11	125.6(3)
C13A	P1A	C1A	108.24(16)	O16	C16	C16^{1}	117.2(2)
C1A	P1A	C7A	110.13(16)	C11	C16	C16^{1}	117.12(19)
C7B	P1B	C19B	106.01(15)	C19A	C20A	C21A	119.2(3)
C7B	P1B	C13B	110.49(15)	C22A	C23A	C24A	120.6(3)
C7B	P1B	C1B	110.99(15)	C13B	C14B	C15B	119.4(4)
C13B	P1B	C19B	113.04(15)	C12B	C11B	C10B	120.6(3)
C1B	P1B	C19B	109.60(16)	C17A	C18A	C13A	119.9(3)
C1B	P1B	C13B	106.77(16)	C7A	C8A	C9A	119.3(3)
C3	O3	Co1	115.3(2)	C3B	C2B	C1B	119.8(4)
C2	O2	Co1	115.4(2)	C8B	C9B	C10B	120.1(3)
C12	O12	Co1	114.5(2)	C11A	C12A	C7A	119.7(3)
O11A	N11	O11B	126.0(3)	C2	C1	N1	118.2(3)
O11A	N11	C11	117.8(3)	C2	C1	C6	123.5(3)
O11B	N11	C11	116.1(3)	C6	C1	N1	118.0(3)
O4B	N4	O4A	125.7(3)	C15A	C16A	C17A	120.2(3)
O4B	N4	C4	119.5(3)	C5A	C6A	C1A	119.7(4)
O4A	N4	C4	114.8(3)	C23B	C24B	C19B	120.1(3)
O1A	N1	O2B	128.0(4)	O5	C5	C4	124.1(4)
O1A	N1	C1	118.0(3)	O5	C5	C6	117.7(3)
O2B	N1	C1	113.6(4)	C4	C5	C6	118.2(3)
O3	C3	C2	116.2(3)	C4B	C3B	C2B	119.2(4)
O3	C3	C4	125.9(3)	C23A	C22A	C21A	120.0(3)

Atom	Atom	Atom	Angle (˚)	Atom	Atom	Atom	Angle (˚)
C4	C3	C2	117.9(3)	C22A	C21A	C20A	120.4(3)
C20B	C19B	P1B	123.3(3)	C9B	C10B	C11B	120.3(3)
C20B	C19B	C24B	120.0(3)	C23B	C22B	C21B	119.9(3)
C24B	C19B	P1B	116.6(2)	C24B	C23B	C22B	120.2(3)
C24A	C19A	P1A	117.3(2)	C22B	C21B	C20B	120.9(3)
C20A	C19A	P1A	122.3(3)	C16A	C17A	C18A	120.1(3)
C20A	C19A	C24A	120.4(3)	C5B	C6B	C1B	119.0(4)
O2	C2	C3	115.0(3)	C3A	C2A	C1A	119.3(4)
O2	C2	C1	126.3(3)	C17B	C18B	C13B	119.7(4)
C1	C2	C3	118.7(3)	C16A	C15A	C14A	120.7(4)
C11B	C12B	C7B	119.1(3)	C10A	C11A	C12A	120.1(4)
C8A	C7A	P1A	123.4(3)	C9A	C10A	C11A	120.1(4)
C8A	C7A	C12A	119.9(3)	O6	C6	C1	125.5(4)
C12A	C7A	P1A	116.7(3)	O6	C6	C5	117.5(4)
C14A	C13A	P1A	120.9(2)	C1	C6	C5	117.0(3)
C14A	C13A	C18A	119.7(3)	C16B	C15B	C14B	120.2(4)
C18A	C13A	P1A	119.3(3)	C10A	C9A	C8A	120.8(4)
C12B	C7B	P1B	122.6(3)	C5B	C4B	C3B	121.1(4)
C8B	C7B	P1B	117.2(2)	C4A	C5A	C6A	119.6(4)
C8B	C7B	C12B	120.2(3)	C4B	C5B	C6B	120.5(4)
O12	C12	C12[1]	116.16(19)	C4A	C3A	C2A	120.0(4)
O12	C12	C11	125.8(3)	C3A	C4A	C5A	121.1(4)
C11	C12	C12[1]	118.1(2)	C17B	C16B	C15B	120.3(4)
C9B	C8B	C7B	119.7(3)	C16B	C17B	C18B	120.3(4)

[1] 1-x, y, 3/2-z

Table S3. Bond distances (Å) in $(NMe_4)_2[Co_2(NA)_3(H_2O)_4]$ (**2**).

Atom1	Atom2	Distance (Å)	Atom1	Atom2	Distance (Å)
Co1	O16[1]	2.146(4)	C4	C3	1.399(8)
Co1	O1W	2.086(4)	C4	C5	1.426(8)
Co1	O3	2.122(4)	C11	C16	1.414(8)
Co1	O2	2.048(4)	C11	C12	1.386(8)
Co1	O12	2.087(4)	C11	N11	1.598(6)
Co1	O2W	2.034(4)	C16	C12[1]	1.548(8)
O16	C16	1.244(7)	C2	C3	1.539(8)
O3	C3	1.247(7)	C2	C1	1.381(8)
O2	C2	1.273(7)	C6	C5	1.561(8)
O12	C12	1.252(7)	C6	C1	1.406(8)
O5	C5	1.223(7)	C1	N1	1.764(8)
O6	C6	1.233(7)	C1	O1C	1.514(10)
N4	O4A	1.199(6)	O4B	O4C	0.762(14)
N4	C4	1.500(7)	N11	O11A	1.177(5)
N4	O4B	1.241(7)	N11	O1	1.185(6)
N4	O4C	1.212(9)	N1	O1B	0.913(9)
N1T	C2T	1.488(8)	N1	O1A	1.351(11)
N1T	C4T	1.492(9)	O1B	O1C	1.208(12)
N1T	C1T	1.494(9)	O1A	O1C	1.188(12)
N1T	C3T	1.501(9)			

[1] 2-x, 1-y, 1-z

Table S4. Bond angles (°) in $(NMe_4)_2[Co_2(NA)_3(H_2O)_4]$ (**2**).

Atom1	Atom2	Atom3	Angle (°)	Atom1	Atom2	Atom3	Angle (°)
O1W	Co1	O16[1]	86.92(16)	O12	C12	C11	126.4(5)
O1W	Co1	O3	83.11(16)	O12	C12	C16[1]	113.9(5)
O1W	Co1	O12	95.69(15)	C11	C12	C16[1]	119.7(5)
O3	Co1	O16[1]	118.41(15)	O2	C2	C3	114.7(5)
O2	Co1	O16[1]	164.32(16)	O2	C2	C1	124.4(5)
O2	Co1	O1W	94.35(16)	C1	C2	C3	120.9(5)
O2	Co1	O3	77.23(15)	O3	C3	C4	126.2(5)
O2	Co1	O12	89.07(15)	O3	C3	C2	115.2(5)
O12	Co1	O16[1]	75.26(15)	C4	C3	C2	118.6(5)
O12	Co1	O3	166.09(16)	O6	C6	C5	117.3(5)
O2W	Co1	O16[1]	89.27(16)	O6	C6	C1	123.4(6)
O2W	Co1	O1W	164.10(17)	C1	C6	C5	119.2(5)
O2W	Co1	O3	85.15(16)	O5	C5	C4	128.0(5)
O2W	Co1	O2	93.48(16)	O5	C5	C6	113.9(5)
O2W	Co1	O12	98.26(16)	C4	C5	C6	118.0(5)
C16	O16	Co1[1]	116.5(4)	C2	C1	C6	121.3(5)
C3	O3	Co1	115.5(4)	C2	C1	N1	119.1(5)
C2	O2	Co1	117.3(4)	C2	C1	O1C	120.8(5)
C12	O12	Co1	119.1(4)	C6	C1	N1	119.1(5)
O4A	N4	C4	116.9(5)	C6	C1	O1C	117.7(5)
O4A	N4	O4B	128.4(6)	O1C	C1	N1	10.2(4)
O4A	N4	O4C	116.0(9)	O4C	O4B	N4	69.8(8)
O4B	N4	C4	114.1(5)	O4B	O4C	N4	74.0(9)
O4C	N4	C4	119.1(8)	O11A	N11	C11	113.5(4)
O4C	N4	O4B	36.2(7)	O11A	N11	O1	134.2(5)
C3	C4	N4	119.6(5)	O1	N11	C11	112.3(4)
C3	C4	C5	121.9(5)	O1B	N1	C1	112.8(7)
C5	C4	N4	118.5(5)	O1B	N1	O1A	145.1(8)
C16	C11	N11	119.2(5)	O1A	N1	C1	95.3(6)
C12	C11	C16	121.8(5)	N1	O1B	O1C	13.3(6)
C12	C11	N11	119.0(4)	O1C	O1A	N1	15.7(5)
O16	C16	C11	126.3(5)	O1B	O1C	C1	113.1(7)
O16	C16	C12[1]	115.2(5)	O1A	O1C	C1	117.7(8)
C11	C16	C12[1]	118.5(5)	O1A	O1C	O1B	129.1(10)

[1] 2-x, 1-y, 1-z

Table S5. Bond distances (Å) in $(NPr_4)_2[Co_3(NA)_4(H_2O)_6]$ (**3**).

**Atom**	**Atom**	**Length (Å)**	**Atom**	**Atom**	**Length (Å)**
Co1	O13	2.090(3)	O6	C6	1.227(5)
Co1	O2	2.089(3)	O1A	N1	1.240(4)
Co1	O12	2.090(3)	O5	C5	1.237(5)
Co1	O12W	2.137(3)	N1	C7	1.445(5)
Co1	O3	2.044(3)	C16	C11	1.396(5)
Co1	O11W	2.046(3)	C16	C15	1.539(5)
Co2	O21W[1]	2.102(3)	C13	C14	1.418(5)
Co2	O21W	2.102(3)	C13	C12	1.538(5)
Co2	O15[1]	2.077(3)	C14	C15	1.407(5)
Co2	O15	2.077(3)	C12	C11	1.408(5)
Co2	O16	2.044(3)	C3	C2	1.529(5)
Co2	O16[1]	2.044(3)	C3	C4	1.395(5)
O13	C13	1.247(5)	C2	C7	1.404(5)
O2	C2	1.256(5)	N1T	C7T	1.526(6)
O12	C12	1.247(5)	N1T	C4T	1.511(6)
N11	O11B	1.189(4)	N1T	C1T	1.501(6)
N11	C11	1.565(4)	N1T	C10T	1.525(6)
N11	O11A	1.197(8)	C7	C6	1.443(5)
N11	O11	1.104(11)	C4	C5	1.404(6)
O15	C15	1.255(5)	C6	C5	1.542(6)
O16	C16	1.251(5)	O11A	O11	0.600(13)
N14	O14B	1.187(4)	C7T	C8T	1.504(7)
N14	C14	1.535(4)	C4T	C5T	1.500(8)
N14	O14A	1.185(5)	C1T	C2T	1.509(7)
O3	C3	1.261(5)	C8T	C9T	1.521(8)
N4	C4	1.562(5)	C10T	C11T	1.530(8)
N4	O4A	1.063(5)	C2T	C3T	1.513(8)
N4	O4B	1.262(5)	C6T	C5T	1.501(7)
O1B	N1	1.213(4)	C11T	C12T	1.525(10)

[1] 2-x, 1-y, 1-z

Table S6. Bond angles (º) in $(NPr_4)_2[Co_3(NA)_4(H_2O)_6]$ (**3**).

**Atom**	**Atom**	**Atom**	**Angle (º)**	**Atom**	**Atom**	**Atom**	**Angle (º)**
O13	Co1	O12	76.25(10)	O16	C16	C15	115.3(3)
O13	Co1	O12W	86.98(11)	C11	C16	C15	119.6(3)
O2	Co1	O13	97.60(10)	O13	C13	C14	126.0(3)
O2	Co1	O12	173.45(10)	O13	C13	C12	114.2(3)
O2	Co1	O12W	87.81(11)	C14	C13	C12	119.8(3)
O12	Co1	O12W	89.61(11)	C13	C14	N14	119.8(3)
O3	Co1	O13	174.53(10)	C15	C14	N14	120.0(3)
O3	Co1	O2	77.42(10)	C15	C14	C13	120.2(3)
O3	Co1	O12	108.64(10)	O12	C12	C13	115.2(3)
O3	Co1	O12W	90.56(11)	O12	C12	C11	125.6(3)
O3	Co1	O11W	91.02(12)	C11	C12	C13	119.2(3)
O11W	Co1	O13	91.58(12)	O3	C3	C2	116.0(3)
O11W	Co1	O2	94.03(11)	O3	C3	C4	125.2(4)
O11W	Co1	O12	88.43(12)	C4	C3	C2	118.7(3)
O11W	Co1	O12W	177.79(10)	O2	C2	C3	113.4(3)
O21W	Co2	O21W[1]	180.00(10)	O2	C2	C7	127.0(4)
O15	Co2	O21W[1]	91.60(11)	C7	C2	C3	119.6(3)
O15	Co2	O21W	88.40(11)	C16	C11	N11	119.1(3)
O15[1]	Co2	O21W	91.60(11)	C16	C11	C12	121.4(3)
O15[1]	Co2	O21W[1]	88.40(11)	C12	C11	N11	119.5(3)
O15	Co2	O15[1]	180.0	O15	C15	C16	114.2(3)
O16[1]	Co2	O21W	89.62(10)	O15	C15	C14	125.9(3)
O16	Co2	O21W[1]	89.61(11)	C14	C15	C16	119.9(3)
O16[1]	Co2	O21W[1]	90.39(11)	C4T	N1T	C7T	111.6(4)
O16	Co2	O21W	90.38(11)	C4T	N1T	C10T	110.3(4)
O16	Co2	O15	77.84(10)	C1T	N1T	C7T	111.8(4)
O16	Co2	O15[1]	102.16(10)	C1T	N1T	C4T	106.6(3)
O16[1]	Co2	O15[1]	77.84(10)	C1T	N1T	C10T	110.4(4)
O16[1]	Co2	O15	102.16(10)	C10T	N1T	C7T	106.1(3)
O16	Co2	O16[1]	180.00(16)	C2	C7	N1	119.9(3)
C13	O13	Co1	117.4(2)	C2	C7	C6	120.9(3)
C2	O2	Co1	116.4(2)	C6	C7	N1	119.3(3)
C12	O12	Co1	116.8(2)	C3	C4	N4	119.0(3)
O11B	N11	C11	113.3(3)	C3	C4	C5	122.8(4)
O11B	N11	O11A	133.8(4)	C5	C4	N4	118.1(3)
O11A	N11	C11	111.2(4)	O6	C6	C7	127.4(4)
O11	N11	O11B	125.6(7)	O6	C6	C5	114.4(3)
O11	N11	C11	117.8(6)	C7	C6	C5	118.2(3)
O11	N11	O11A	29.9(7)	O5	C5	C4	124.2(4)
C15	O15	Co2	116.0(2)	O5	C5	C6	117.3(3)
C16	O16	Co2	116.6(2)	C4	C5	C6	118.4(3)
O14B	N14	C14	115.6(3)	O11	O11A	N11	66.4(14)

Atom	Atom	Atom	Angle (º)	Atom	Atom	Atom	Angle (º)
O14A	N14	O14B	127.5(3)	C8T	C7T	N1T	116.4(4)
O14A	N14	C14	115.7(3)	C5T	C4T	N1T	116.1(4)
C3	O3	Co1	115.9(2)	N1T	C1T	C2T	116.0(4)
O4A	N4	C4	120.0(4)	C7T	C8T	C9T	108.6(5)
O4A	N4	O4B	129.8(5)	N1T	C10T	C11T	116.4(4)
O4B	N4	C4	109.8(4)	C1T	C2T	C3T	109.2(5)
O1B	N1	O1A	122.1(3)	C12T	C11T	C10T	108.1(5)
O1B	N1	C7	119.6(3)	C4T	C5T	C6T	108.8(5)
O1A	N1	C7	118.0(3)	O11A	O11	N11	83.8(17)
O16	C16	C11	125.1(3)				

[1] 2-x, 1-y, 1-z

Table S7. Bond distances (Å) in [Co(NA)(H_2O)$_2$] (**4**).

Atom	Atom	Length (Å)
Co1	O6[1]	2.115(3)
Co1	O6[2]	2.115(3)
Co1	O2[3]	2.070(3)
Co1	O2	2.070(3)
Co1	O11W	2.064(3)
Co1	O11W[3]	2.064(3)
O6	C6	1.240(5)
O2	C2	1.251(5)
N1	O1A	1.221(4)
N1	O1B	1.183(5)
N1	C1	1.508(5)
C6	C2[1]	1.549(5)
C6	C1	1.402(5)
C2	C1	1.395(6)

[1] 1/2-x, 3/2-y, 3/2-z; [2] x, 3/2-y, -1/2+z; [3] 1/2-x, y, 1-z

Table S8. Bond angles (º) in $[Co(NA)(H_2O)_2]$ (**4**).

Atom	Atom	Atom	Angle (º)	Atom	Atom	Atom	Angle (º)
O6^1	Co1	O6^2	98.08(16)	C6	O6	Co1^2	114.8(3)
O2	Co1	O6^2	76.95(11)	C2	O2	Co1	116.6(2)
O2^3	Co1	O6^2	91.80(11)	O1A	N1	C1	114.1(3)
O2^3	Co1	O6^1	76.95(11)	O1B	N1	O1A	127.9(3)
O2	Co1	O6^1	91.80(11)	O1B	N1	C1	118.0(3)
O2^3	Co1	O2	162.94(18)	O6	C6	C2^2	115.3(3)
O11W^3	Co1	O6^1	87.50(13)	O6	C6	C1	126.8(4)
O11W^3	Co1	O6^2	172.39(11)	C1	C6	C2^2	117.8(3)
O11W	Co1	O6^2	87.50(12)	O2	C2	C6^2	114.7(3)
O11W	Co1	O6^1	172.39(11)	O2	C2	C1	126.0(4)
O11W^3	Co1	O2^3	94.52(12)	C1	C2	C6^2	119.3(3)
O11W^3	Co1	O2	97.80(12)	C6	C1	N1	118.6(3)
O11W	Co1	O2	94.51(12)	C2	C1	N1	118.7(3)
O11W	Co1	O2^3	97.80(12)	C2	C1	C6	122.6(3)
O11W^3	Co1	O11W	87.44(18)				

^{1} x, 3/2-y, -1/2+z; ^{2} 1/2-x, 3/2-y, 3/2-z; ^{3} 1/2-x, y, 1-z

Table S9. Continuous SHAPE measurement (CShM) values of the five possible coordination geometries for the Co^{II} ion with coordination number six in compounds **1-4**.[1,2]

Geometry	Symmetry	1	2	3A	3B	4
HP-6	D_{6h}	26.788	29.287	27.516	28.149	31.579
PPY-6	C_{5v}	26.648	20.049	26.330	27.905	23.861
**OC-6**	**O_h**	**1.112**	**2.713**	**1.005**	**0.771**	**1.201**
TPR-6	D_{3h}	14.928	9.626	14.354	15.821	11.939
JPPY-6	C_{5v}	29.653	23.680	29.405	30.831	27.598

HP-6 = Hexagon, PPY-6 = Pentagonal pyramid, OC-6 = Octahedron, TPR-6 = Trigonal prism and JPPY-6 = Johnson pentagonal pyramid J2. The lowest values are indicated in bold.

Table S10. Oxidation state (OE) of the cobalt ions in compounds **1-4** determined with the Bond Valence Sum calculations assuming +2 and +3 EO.[3]

Assumed OE	1	2	3-Co1	3-Co2	4
+2	2.034	2.038	2.056	2.099	2.050
+3	1.786	1.790	1.806	1.844	1.801

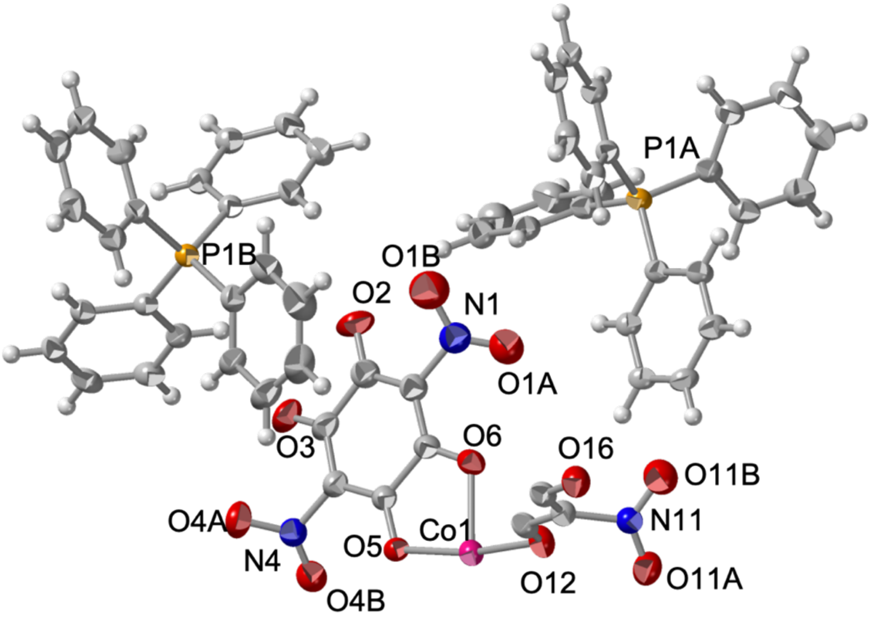

Figure S4. *ORTEP view of the asymmetric unit of compound $(PPh_4)_4[Co(NA)_3]$ (**1**) with the labelling scheme. Ellipsoids drawn at 50 % probability.*

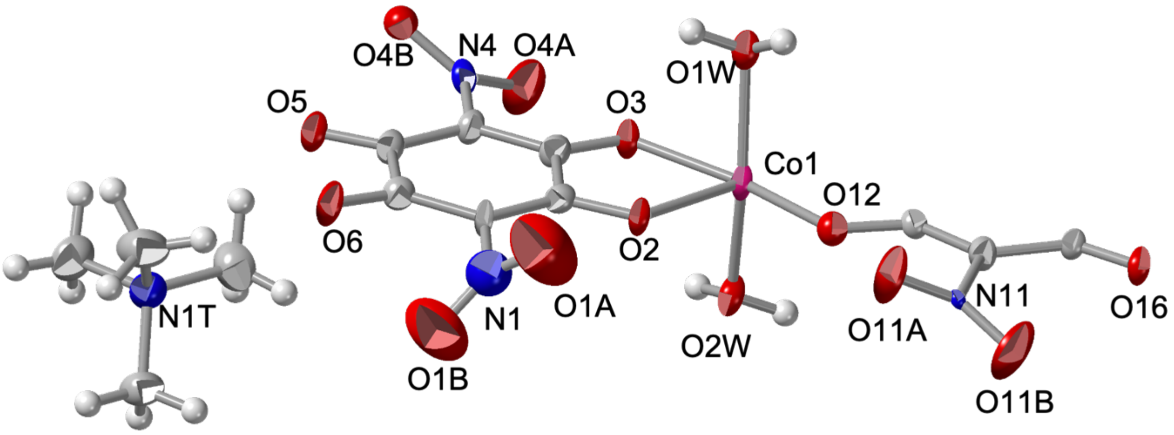

Figure S5. *ORTEP view of the asymmetric unit of compound $(NMe_4)_2[Co_2(NA)_3(H_2O)_4]$ (**2**) with the labelling scheme. Ellipsoids drawn at 50 % probability.*

Table S11. Interdimer H-bond parameters in $(NMe_4)_2[Co_2(NA)_3(H_2O)_4]$ (**2**).

**Type**	**Atoms**	**D-H (Å)**	**H···A (Å)**	**D···A (A)**	**<D-H···A> (º)**
a	O2W-H2WB···O5	0.9160	1.8706	2.768	165.82
b	O1W-H1WA···O2	0.9143	1.8782	2.758	160.79
c	O2W-H2WA···O6	0.9170	1.8849	2.645	138.93
d	O1W-H1WB···O11A	0.9146	2.1541	2.934	142.67
e	O1W-H1WB···O12	0.9146	2.1764	2.930	139.18

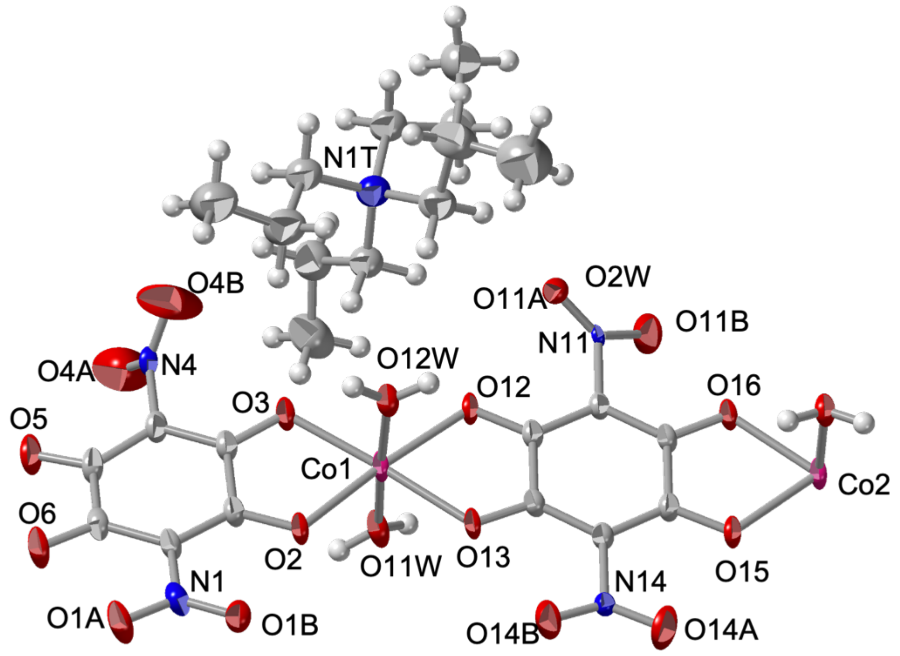

Figure S6. *ORTEP view of the asymmetric unit of compound $(NPr_4)_2[Co_3(NA)_4(H_2O)_6]$ (**3**) with the labelling scheme. Ellipsoids drawn at 50 % probability.*

Table S12. Interdimer H-bond parameters in $(NPr_4)_2[Co_3(NA)_4(H_2O)_6]$ (**3**).

Type	Atoms	D-H (Å)	H···A (Å)	D···A (A)	<D-H···A> (º)
a	O11W-H11A···O5	0.8703	1.9657	2.749	149.01
b	O11W-H11B···O6	0.8697	1.9483	2.806	168.68
c	O21W-H21A···O2	0.8756	1.9781	2.801	155.97
d	O21W-H21B···O13	0.8742	2.0963	2.891	150.83

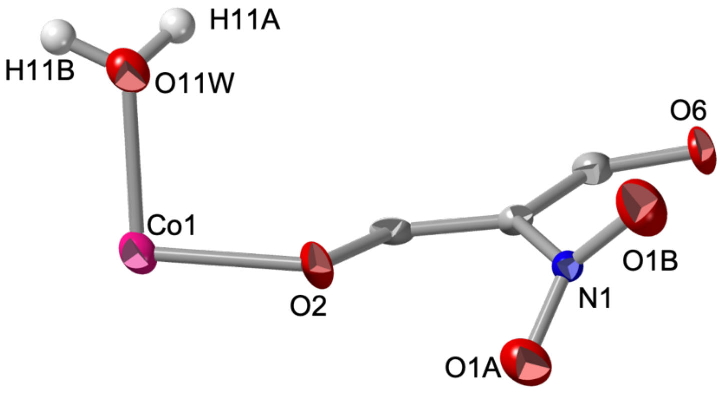

Figure S7. *ORTEP view of the asymmetric unit of compound $[Co(NA)(H_2O)_2]$ (**4**) with the labelling scheme. Ellipsoids drawn at 80 % probability.*

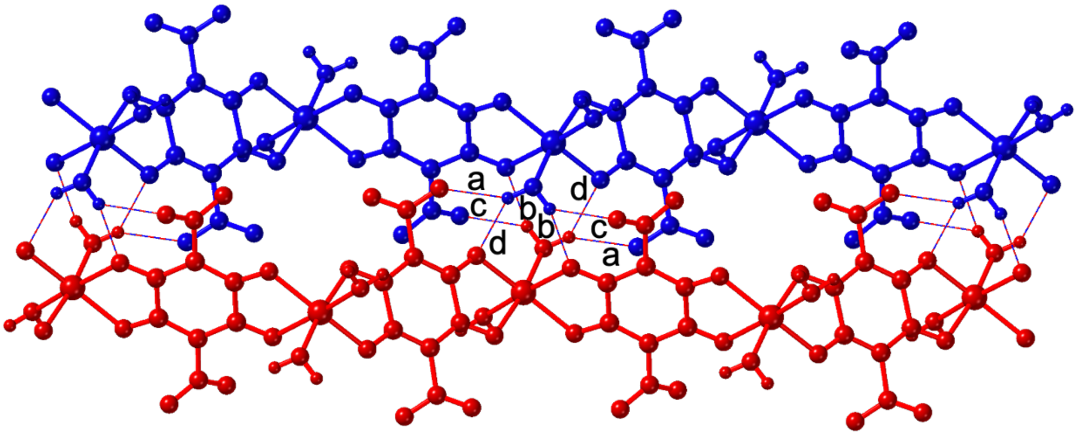

Figure S8*. View of two chains (in red and blue) in compound [Co(NA)(H_2O)$_2$] (**4**) showing the inter-chain H-bonds as thin red and blue lines. Letters a-d indicate the H-bond type (see Table S13).*

Table S13. Inter-chain H-bond parameters in [Co(NA)(H_2O)$_2$] (**4**).

Type	Atoms	D-H (Å)	H···A (Å)	D···A (A)	<D-H···A> (º)
a	O11W-H11A···O1A	0.8702	2.1719	2.933	145.82
b	O11W-H11B···O6	0.8701	2.2174	2.888	139.62
c	O11W-H11B···O1B	0.8701	2.2896	2.978	136.11
d	O11W-H11A···O2	0.8702	2.3669	3.037	138.35

References

1. Alvarez, S. Distortion Pathways of Transition Metal Coordination Polyhedra Induced by Chelating Topology. *Chem. Rev.* **2015**, *115*, 13447–13483.

2. Alvarez, S.; Avnir, D.; Llunell, M.; Pinsky, M. Continuous symmetry maps and shape classification. The case of six-coordinated metal compounds. *New J. Chem.* **2002**, *26*, 996–1009.

3. Wood, R. M.; Palenik, G. J. Bond valence sums in coordination chemistry. A simple method for calculating the oxidation state of cobalt in complexes containing only Co−O bonds. *Inorg. Chem.* **1998**, *37*, 4149–4151.
